# IGF1, serum glucose, and retinopathy of prematurity in extremely preterm infants

**DOI:** 10.1172/jci.insight.140363

**Published:** 2020-10-02

**Authors:** Bertan Cakir, William Hellström, Yohei Tomita, Zhongjie Fu, Raffael Liegl, Anna Winberg, Ingrid Hansen-Pupp, David Ley, Ann Hellström, Chatarina Löfqvist, Lois E.H. Smith

**Affiliations:** 1 Department of Ophthalmology, Boston Children’s Hospital, Harvard Medical School, Boston, Massachusetts, USA.; 2 Department of Pediatrics, Institute of Clinical Sciences, and; 3 Department of Clinical Neuroscience, Institute of Neuroscience and Physiology, Sahlgrenska Academy, University of Gothenburg, Gothenburg, Sweden.; 4 Örebro University Hospital, Örebro, Sweden.; 5 Department of Pediatrics, Institute of Clinical Sciences Lund, Lund University, Lund, Sweden.; 6 Skane University Hospital, Lund, Sweden.; 7 Institute of Health and Care Sciences, Sahlgrenska Academy, University of Gothenburg, Gothenburg, Sweden.

**Keywords:** Ophthalmology, Glucose metabolism, Insulin signaling, Retinopathy

## Abstract

**BACKGROUND:**

Hyperglycemia, insulin insensitivity, and low IGF1 levels in extremely preterm infants are associated with an increased risk of retinopathy of prematurity (ROP), but the interactions are incompletely understood.

**METHODS:**

In 117 extremely preterm infants, serum glucose levels and parenteral glucose intake were recoded daily in the first postnatal week. Serum IGF1 levels were measured weekly. Mice with oxygen-induced retinopathy alone versus oxygen-induced retinopathy plus streptozotocin-induced hyperglycemia/hypoinsulinemia were assessed for glucose, insulin, IGF1, IGFBP1, and IGFBP3 in blood and liver. Recombinant human IGF1 was injected to assess the effect on glucose and retinopathy.

**RESULTS:**

The highest mean plasma glucose tertile of infants positively correlated with parenteral glucose intake [r_(39)_ = 0.67, *P* < 0.0001]. IGF1 plasma levels were lower in the high tertile compared with those in low and intermediate tertiles at day 28 (*P* = 0.038 and *P* = 0.03). In high versus lower glucose tertiles, ROP was more prevalent (34 of 39 versus 19 of 39) and more severe (ROP stage 3 or higher; 71% versus 32%). In oxygen-induced retinopathy, hyperglycemia/hypoinsulinemia decreased liver IGF1 expression (*P* < 0.0001); rh-IGF1 treatment improved normal vascular regrowth (*P* = 0.027) and reduced neovascularization (*P* < 0.0001).

**CONCLUSION:**

In extremely preterm infants, high early postnatal plasma glucose levels and signs of insulin insensitivity were associated with lower IGF1 levels and increased ROP severity. In a hyperglycemia retinopathy mouse model, decreased insulin signaling suppressed liver IGF1 production, lowered serum IGF1 levels, and increased neovascularization. IGF1 supplementation improved retinal revascularization and decreased pathological neovascularization. The data support IGF1 as a potential treatment for prevention of ROP.

**TRIAL REGISTRATION:**

ClinicalTrials.gov NCT02760472 (Donna Mega).

**FUNDING:**

This study has been supported by the Swedish Medical Research Council (14940, 4732, 20144-01-3, and 21144-01-3), a Swedish government grant (ALFGB2770), Lund medical faculty grants (ALFL, 11615 and 11601), the Skåne Council Foundation for Research and Development, the Linnéa and Josef Carlsson Foundation, the Knut and Alice Wallenberg Foundation, the NIH/National Eye Institute (EY022275, EY017017, EY017017-13S1, and P01 HD18655), European Commission FP7 project 305485 PREVENT-ROP, Deutsche Forschungsgemeinschaft (CA-1940/1-1), and Stiftelsen De Blindas Vänner.

## Abstract 2

In extremely preterm infants, high early postnatal plasma glucose levels and signs of insulin insensitivity were associated with lower IGF1 levels and increased retinopathy of prematurity severity.

## Introduction

Hyperglycemia with insulin insensitivity in the first few postnatal weeks of life is a common complication in extremely preterm infants. In addition, infants born extremely preterm with insulin resistance have an increased risk of cardiovascular morbidity later in life (1), possibly secondary to altered metabolic programming during the neonatal period.

During the first week of life, 80% of infants with birth weights of less than 1500 g develop hyperglycemia, which is associated with increased mortality and morbidity, such as retinopathy of prematurity (ROP) and bronchopulmonary dysplasia (2–5), as well as impaired long-term growth (6). Extremely low birth weight infants on total parenteral nutrition may have continuous hepatic gluconeogenesis, which is unaffected by infusion rates of glucose or concentrations of glucose or insulin (7).

Hyperglycemia in preterm infants has also been associated with low insulin growth factor 1 (IGF1) levels (8). IGF1 is essential for critical aspects of prenatal and postnatal development. Following preterm birth, IGF1 serum levels fall rapidly to levels 5-fold lower than those in full-term infants (~10 ng/mL in preterm infants vs. >50 ng/mL at the same gestational age in utero) and remain low for the first weeks of life (9–13). Persistent low IGF1 serum levels after preterm birth have been associated with poor postnatal development as well as neonatal morbidities, such as intraventricular hemorrhage, bronchopulmonary dysplasia, necrotizing enterocolitis, and ROP (13–18). Therefore, identifying premature infants who have low IGF1 levels and who fail to increase their IGF1 production postnatally could have important clinical implications.

Current treatment strategies for ROP address end-stage vasoproliferative diseases (phase II), such as intravitreal anti-VEGF injections and laser photocoagulation. These interventions are invasive and can lead to serious complications. Intravitreal anti-VEGF injection results in a concerning suppression of systemic levels of VEGF, which may suppress normal organ vascularization. Therefore, treatments targeted at phase I ROP to improve physiologic vascularization of the retina and prevent neovascularization are needed.

Even though independent associations among hyperglycemia, ROP, and IGF1 have been described, a more detailed understanding of ROP risk, with specific levels of hyperglycemia during different postnatal weeks, the contribution of insulin insensitivity, and the role of IGF1, is needed. To better determine these interassociations, we examined the relationship between intravenously administered glucose and levels of serum glucose (reflecting insulin sensitivity and insufficient insulin production) and the effect of plasma glucose level on IGF1 levels and on ROP development in 2 prospective longitudinal clinical studies in extremely preterm infants. Experimentally, we examined these interactions in a hyperglycemia/hypoinsulinemia oxygen-induced retinopathy mouse model.

## Results

### Clinical data

#### Clinical characteristics of study population.

**Figure 1:**
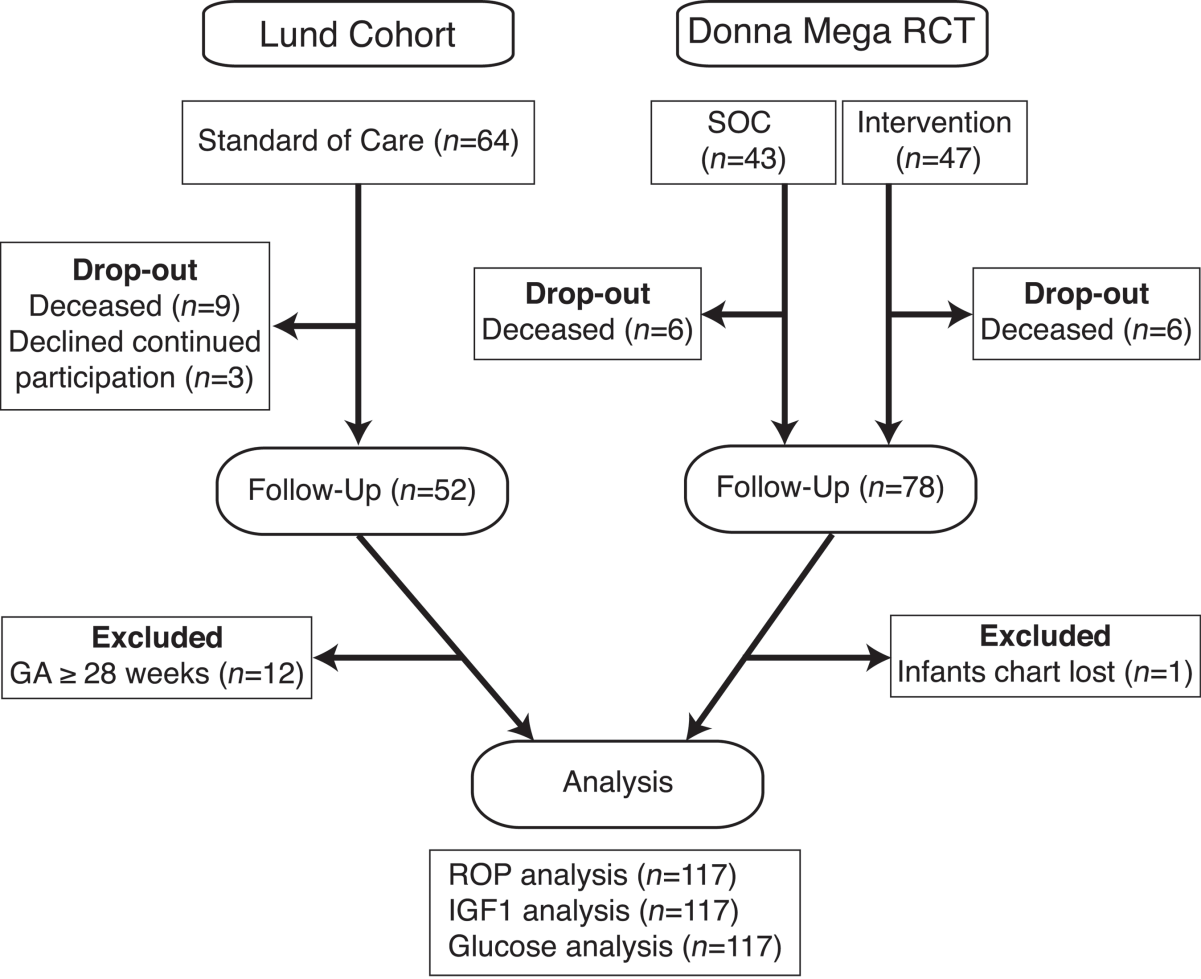
Flow chart for the selection of study participants. A total of 64 patients participated in the Lund prospective longitudinal observation study, and a total of 90 patients were included in the randomized controlled Donna Mega trial. In each study 12 patients were lost to follow-up (drop out). In the Lund cohort a total of 12 infants were excluded from analysis due to advanced gestational age (GA ≥28 weeks). Only 1 patient was excluded from the Donna Mega randomized controlled trial due to loss of records. SOC, standard of care.

A total of 117 extremely preterm infants with gestational age at birth <28 weeks were included in the clinical analysis (Figure 1). The mean gestational age and mean birth weight were 25.4 weeks (range, 22.71–27.86 weeks) and 780 g (range, 348–1260 g). Of the included 117 patients, 79 developed ROP (67.5%), 73 (62%) developed bronchopulmonary dysplasia, and 43 (37%) developed intraventricular hemorrhage (Table 1).

#### Glucose homeostasis during first week of life.

In our cohort of extremely preterm infants, 75 of 117 (64%) had at least 1 episode and 41 of 117 (35%) had at least 2 episodes of hyperglycemia (>10 mmol/L) in the first 7 postnatal days. Mean plasma glucose concentration in the first week of life correlated negatively with gestational age at birth [r_(117)_ = –0.648, *P* < 0.0001] and with birth weight [r_(117)_ = –0.681, *P* < 0.0001].

**Figure 2:**
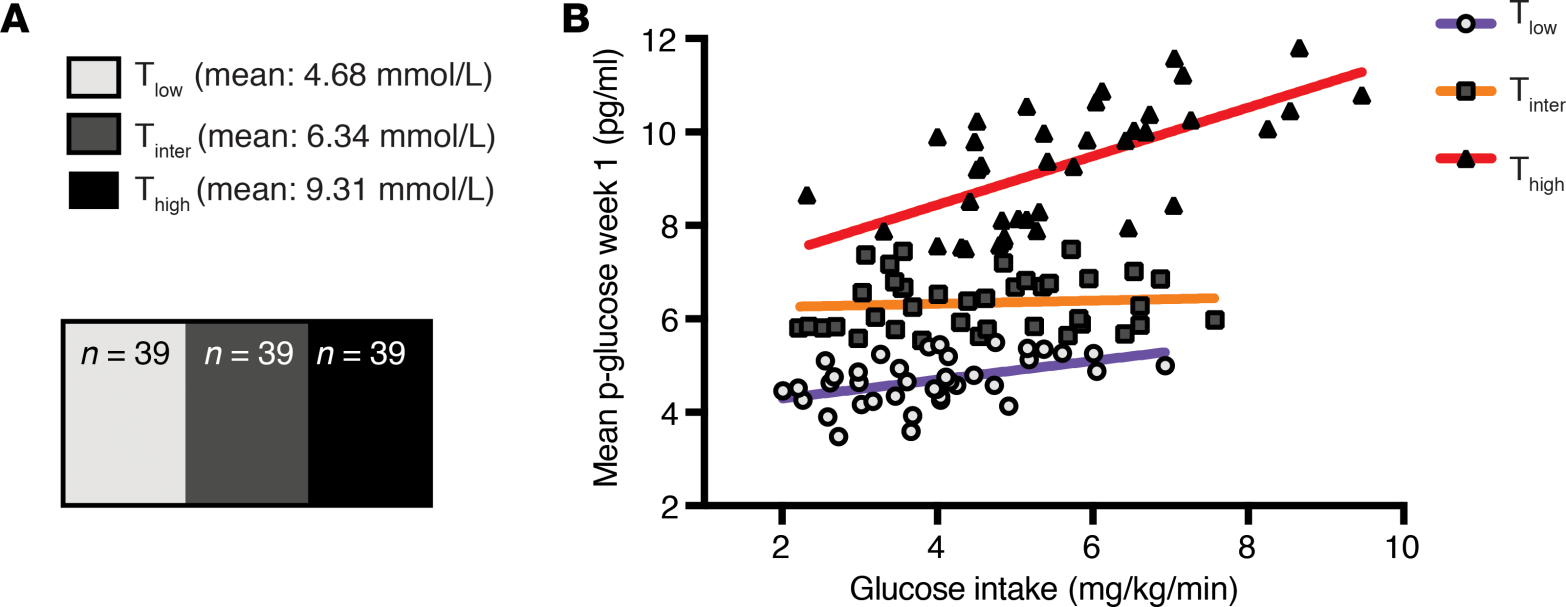
Hyperglycemia in the first week of life is associated with poor glucose control. (**A**) Illustration of glucose tertiles constructed using mean plasma glucose levels during the first week of life. (**B**) Correlation between mean plasma glucose and parenteral glucose intake in the first week of life (*n* = 39 each group). Spearman’s correlation (r) for the relationship between glucose levels and glucose intake within the respective glucose tertiles were 0.46 for T_low_ (*P* = 0.003), 0.16 for T_intermediate_ (*P* = 0.32), and 0.67 for T_high_ (*P* < 0.0001).The colored lines indicate the slope of the linear regression (red, T_high_; orange, T_intermediate_; purple, T_low_).

To further investigate the correlation between hyperglycemia and ROP, we divided our cohort into glucose tertiles. Cutoff points of plasma glucose tertiles (39 infants/tertile) were constructed using mean plasma glucose levels during the first week of life of life: T_low_ 4.68 mmol/L (range, 3.48–5.50 mmol/L); T_intermediate_ 6.34 mmol/L (range, 5.54–7.49 mmol/L), and T_high_ 9.31 mmol/L (7.51–11.81 mmol/L) (Figure 2A).

We found a positive correlation between total parenteral glucose intake and plasma glucose levels for the whole cohort [r_(117)_ = 0.55, *P* < 0.0001]. Spearman’s correlation (r) for the relationship between glucose levels and glucose intake within the respective glucose tertiles were 0.46 for T_low_ (*P* = 0.003), 0.16 for T_intermediate_ (*P* = 0.32), and 0.67 for T_high_ (*P* < 0.0001). The slope was highest in T_high_ (*y* = 0.52 × *x* + 6.36) compared with T_intermediate_ (*y* = 0.03 × *x* + 6.19) and T_low_ (*y* = 0.2 × *x* + 3.9) (Figure 2B).

#### IGF1 concentrations in relation to first week postnatal day glucose tertiles.

**Figure 3:**
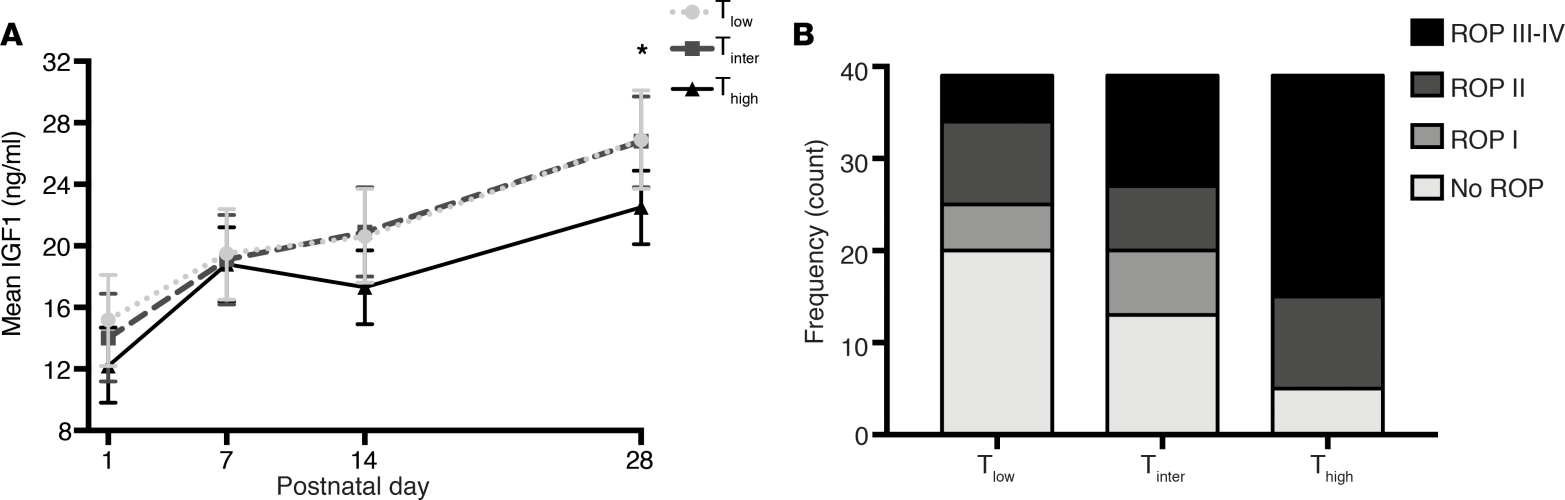
Plasma IGF1 levels from P1 to P28 and ROP frequency. (**A**) Mean plasma IGF1 levels from P1 to P28. The high glucose tertile group showed lower mean IGF1 levels through P1–P28 compared with T_low_ and T_intermediate_, with a significant difference compared with T_low_ and T_intermediate_ at P28 (*P* = 0.038 and *P* = 0.030, mixed model regression). (**B**) Relationship between glucose tertile and frequency of retinopathy of prematurity (ROP) stages. Severity and frequency of ROP was higher in the high glucose tertile group compared with the intermediate and low glucose tertile group (*P* < 0.0001). *P < 0.05, Mantel-Haenszel χ^2^ test. Error bars represent 95% CI.

Mean plasma IGF1 levels at P1, P7, and P14 were decreased in the T_high_ plasma glucose tertile compared with the intermediate and low plasma glucose tertile but not statistically significant. At P28, infants in the T_high_ plasma glucose tertile had significantly lower mean IGF1 levels than infants in both the T_low_ and T_intermediate_ tertiles (Figure 3A). The least square mean (95% CI) per glucose tertile and difference in least square mean between glucose tertiles assessed by mixed model regression analysis is detailed in Table 2.

#### Glucose homeostasis in relation to ROP.

The relationships between plasma glucose tertiles and ROP outcome are shown in Figure 3B. We noted that the frequencies and severity of ROP had a tendency to increase in T_intermediate_ and T_high_ plasma glucose tertile groups. ROP frequency was also evaluated against the commonly used glucose threshold for hyperglycemia (19) (>8.3 mmol/L) and hyperglycemia needing treatment (>10 mmol/L). In infants with severe ROP, 97.6% (40 of 41, *P* < 0.0001) had hyperglycemia and 85.4% (35 of 41, *P* < 0.0001) had hyperglycemia needing treatment.

### Experimental data

#### The neonatal hyperglycemia oxygen-induced model.

**Figure 4:**
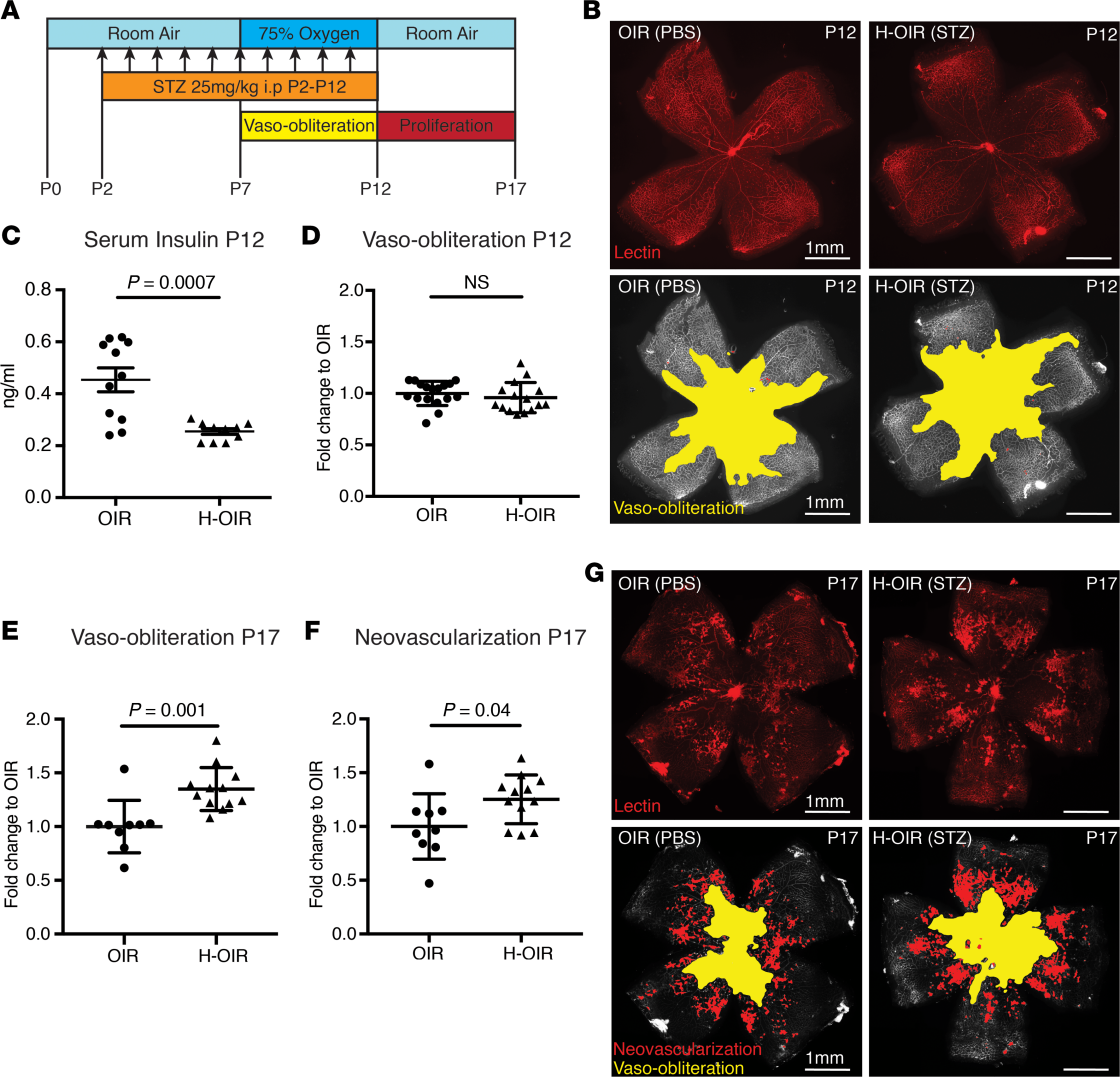
The neonatal hyperglycemia/hypoinsulinemia oxygen-induced retinopathy model. (**A**) Scheme of neonatal hyperglycemia oxygen-induced retinopathy model (hyperglycemia/hypoinsulinemia oxygen-induced retinopathy [H-OIR]) vs. OIR control: C57BL/6 mouse pups were exposed to 75% oxygen from P7 to P12 to induce central vaso-obliteration (VO) and returned to room air at P12–P17, which was characterized by vascular regrowth and later by neovascular tuft formation (NV). To induce hyperglycemia, streptozotocin or PBS was injected daily (25 μg per gram body weight) from P2 to P12. (**B**) Top: Representative retinal whole-mount images at P12 stained with lectin (OIR, left; H-OIR, right). Bottom: Images after fully automated deep-learning segmentation. The yellow outlines the VO area. Scale bar: 1 mm. (**C**) Serum insulin levels were decreased at P12 in H-OIR mice compared with OIR controls (*n* = 10 for both groups; *P* = 0.0007). (**D**) There was no difference in VO between H-OIR and OIR mice at P12 (*P* = 0.39; *n* = 18 eyes for OIR and *n* = 16 eyes for H-OIR). (**E**) There was a significant difference in VO between H-OIR and OIR mice at P17 (*P* = 0.001; *n* = 9 eyes for OIR and *n* = 12 eyes for H-OIR). (**F**) There was a significant difference in NV between H-OIR and OIR mice at P17 (*P* = 0.049; *n* = 9 eyes for OIR and *n* = 12 eyes for H-OIR). (**G**) Top: Representative lectin-stained retinal whole-mount images at P17 (OIR, left; H-OIR, right). Bottom: Images after fully automated deep-learning segmentation. The yellow outlines the VO, and the red the NV area. Error bars represent mean ± SD. Unpaired *t* test was used for statistical analysis.

**Figure 5:**
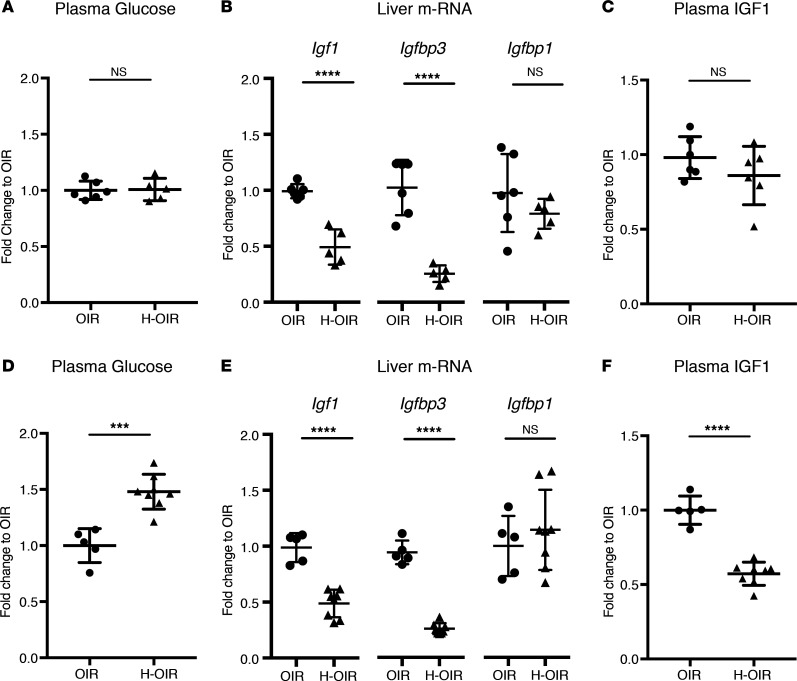
IGF1 and glucose expression profile in the neonatal hyperglycemia/hypoinsulinemia oxygen-induced retinopathy model. (**A**) Plasma glucose levels at P12 were unchanged between oxygen-induced retinopathy (OIR) controls (PBS) and hyperglycemia OIR (H-OIR) mice (*n* = 5 for OIR and *n* = 8 for H-OIR). (**B**) Liver mRNA expression at P12: *Igf1* and *Igfbp3* was reduced more than 2-fold in the H-OIR group. *Igfbp1* was unchanged. (*n* = 6 for OIR group and *n* = 5 for H-OIR). (**C**) ELISA measurements of plasma IGF1 protein at P12 were unchanged between groups (*n* = 6 in each group). (**D**) Plasma glucose levels at P17 were increased in H-OIR pups (*n* = 5 for OIR and *n* = 8 for H-OIR). (**E**) Liver mRNA expression at P17: *Igf1* and *Igfbp3* were reduced more than 2-fold in the H-OIR group. *Igfbp1* was unchanged (*n* = 5 for OIR and *n* = 8 for H-OIR). (**F**) ELISA measurements of plasma IGF1 protein at P17 were decreased in H-OIR mice compared with OIR controls (*n* = 5 for OIR and *n* = 8 for H-OIR). ***P < 0.001, ****P < 0.0001. Error bars represent mean ± SD. Unpaired *t* test was used for statistical analysis.

In streptozotocin-injected neonatal hyperglycemia oxygen-induced retinopathy pups, insulin serum levels were significantly decreased at P12 compared with PBS-injected normoglycemic oxygen-induced retinopathy controls (*P* = 0.0007). The avascular retinal area at P12 was unchanged between groups (*P* = 0.39) (Figure 4, A–D). At P17, however, both avascular retinal area and neovascularization were greater in hyperglycemic oxygen-induced retinopathy retinas than in normoglycemic oxygen-induced retinopathy controls (*P* = 0.001 and *P* = 0.04) (Figure 4, E–G). Body weights between groups were comparable at P12 and P17 (*P* = 0.12 and *P* = 0.19). Further, we assessed plasma glucose levels, liver mRNA expression of *Igf1*, insulin-binding protein 3 (*Igfbp3*), and insulin-binding protein 1 (*Igfbp1*), and plasma levels of IGF1 in the neonatal hyperglycemic oxygen-induced retinopathy versus normoglycemic oxygen-induced retinopathy mice at P12 and P17. Plasma glucose levels were the same in the 2 groups at P12 (Figure 5A). However, *Igf1* and *Igfbp3* mRNA expression in the liver was decreased more than 2-fold in the hyperglycemic oxygen-induced retinopathy pups (both *P* < 0.0001) (Figure 5B). mRNA expression of *Igfbp1*, a negative regulator of IGF1, was the same in each group (Figure 5B). The plasma levels of IGF1 protein were slightly decreased, but not significantly, in hyperglycemic oxygen-induced retinopathy pups (*P* = 0.25, Figure 5C). At P17, plasma glucose levels were significantly increased in the hyperglycemic oxygen-induced retinopathy versus normoglycemic oxygen-induced retinopathy mice (Figure 5D). The mRNA expression profile at P17 was comparable to the expression at P12, with more than a 2-fold decrease of *Igf1* and *Igfbp3* in the hyperglycemic oxygen-induced retinopathy versus normoglycemic oxygen-induced retinopathy groups (both < 0.0001); *Igfbp1* mRNA expression was the same in each group (Figure 5E). The plasma IGF1 protein level at P17 was significantly decreased in the hyperglycemic oxygen-induced retinopathy versus normoglycemic oxygen-induced retinopathy groups (Figure 5F).

#### IGF1 treatment data.

**Figure 6:**
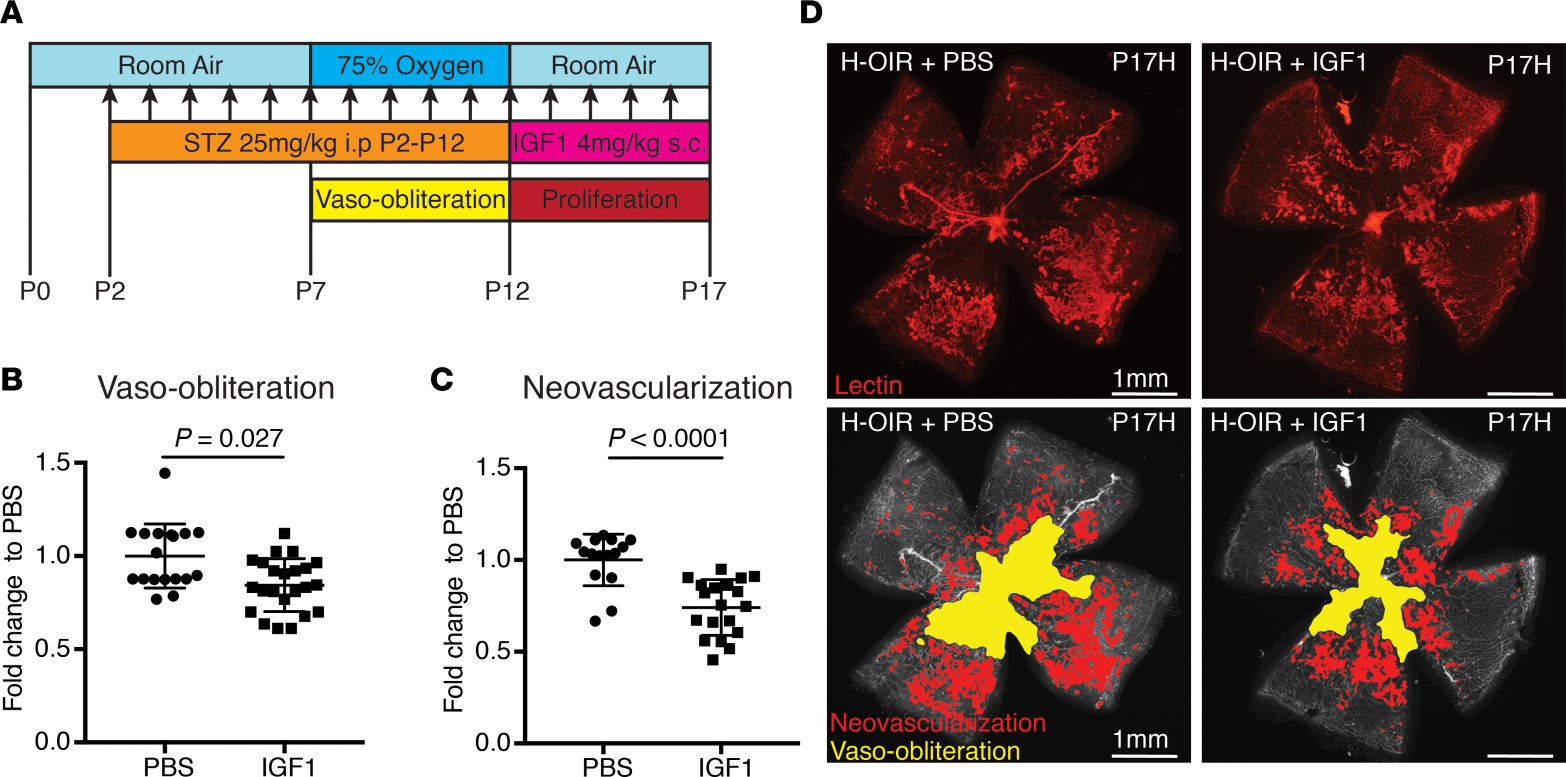
IGF1 reduces neovascularization in the neonatal hyperglycemia/hypoinsulinemia oxygen-induced retinopathy model. (**A**) Scheme of neonatal hyperglycemia oxygen-induced retinopathy (OIR) model (H-OIR): C57BL/6 mouse pups were exposed to 75% oxygen from P7 to P12 to induce central vaso-obliteration (VO) and returned to room air at P12–P17, which was characterized by vascular regrowth and later by neovascular tuft formation (NV). To induce hyperglycemia, streptozotocin (STZ) was injected daily (25 μg per gram body weight) from P2 to P12. IGF1 or control PBS injections were performed twice daily s.c. (4 μg per gram body weight) from P12 to P16. (**B**) Scatter blot of VO at P17: There was a significant difference in VO between IGF1 and PBS at P17 (*P* = 0.027; *n* = 18 eyes for PBS and *n* = 23 eyes for IGF1 in H-OIR). (**C**) Scatter blot of NV at P17: There was a significant difference in NV between IGF1 and PBS at P17 (*P* < 0.0001; *n* = 15 eyes for PBS and *n* = 19 eyes for IGF1). (**D**) Top: Representative retinal whole-mount images at P17 (PBS, left; IGF1, right). Bottom: Images after fully automated deep-learning segmentation. Yellow outlines the VO, and red the NV area. Scale bar: 1 mm. Error bars represent mean ± SD. Unpaired *t* test was used for statistical analysis.

IGF1 treatment of the hyperglycemic oxygen-induced retinopathy mice reduced the retinal avascular retinal area compared with vehicle control at P17 (*P* = 0.027). The neovascularization at P17 was also decreased compared with controls (*P* < 0.0001) (Figure 6). Body weight and glucose were not significantly different between groups (*P* = 0.16 and *P* = 0.39).

## Discussion

During fetal life, insulin is considered to begin to function normally at around 28–30 weeks of gestation (20, 21). Thus, infants born before 28 weeks gestation are at a developmental stage when insulin resistance, although physiologic in utero, puts them at risk for transient hyperglycemia after birth. The incidence of hyperglycemia in the first 7 postnatal days of our cohort (64%, 1 episode, and 35%, 2 episodes) is in line with that in previous reports (2–5). We also found (as have prior studies, refs. 22–24) that, in the first week of life, higher mean plasma glucose concentration correlated with low gestational age as well as low body weight. This suggests that early hyperglycemia may be intrinsic to low gestational age when insulin insensitivity is part of development.

Infants in the high glucose tertile appeared to have the least developed capacity to regulate glucose levels in relation to glucose intake, as shown by the strongest positive correlation between glucose intake and mean plasma glucose levels [r_(39)_ = 0.63, *P* < 0.0001]. The slope was 2.5-fold higher compared with that of the low glucose tertile group (Figure 2B). These results are consistent with increased insulin insensitivity in the high tertile group. Insulin insensitivity in very preterm infants has been attributed to defective processing of proinsulin as well as relative insulin resistance (25).

Infants in the high mean plasma glucose tertile had consistently lower mean IGF1 levels from birth until 28 days of age as compared with infants in both the intermediate and low tertiles, with a significant difference at P28 using mixed model regression analysis (Figure 3A). Infants in the highest glucose tertile with lower IGF1 levels had a profound increase in ROP frequency and severity (Figure 3B). In the high glucose tertile, 34 of 39 infants (87%) developed ROP, of which 71% were severe (stage 3 or higher), compared with only 19 of 39 ROP cases (49%) in the low tertile group, of which the majority were stage 1 and 2. The association of hyperglycemia and comorbidities such as ROP has been previously reported (2–5). A meta-analysis from 2015 (26) identified hyperglycemia in the first week of life as an independent risk factor for ROP with an odds ratio of 4.16 (95%CI 2.09–8.29, I^2^ = 65%, *P* < 0.0001). To validate the use of mean glucose tertiles in our cohort, ROP frequency was also evaluated against the commonly used glucose threshold for hyperglycemia (19) (>8.3 mmol/L) and for hyperglycemia needing treatment (>10 mmol/L). In infants with severe ROP, 97.6% (40 of 41; *P* < 0.0001) had hyperglycemia (>8.3 mmol/L) and 85.4% (35 of 41 *P* < 0.0001) had hyperglycemia needing treatment (>10 mmol/L).

In line with the clinical data, the neonatal hyperglycemia/hypoinsulinemia oxygen-induced retinopathy mouse model showed more severe retinopathy, with increased avascular area and increased neovascularization at P17. In oxygen-induced retinopathy mice, the addition of hyperglycemia/hypoinsulinemia (Figure 4, A–C) did not affect oxygen-induced vessel loss at P12 but suppressed normal vascular regrowth between P12 and P17. Increased avascular area has been shown to lead to more hypoxia with upregulation of oxygen-regulated angiogenic factors such as VEGF causing increased pathological neovascularization at P17.

The increased prevalence of any ROP and of severe ROP seen clinically and experimentally is possibly explained by decreased IGF1 expression mediated through decreased insulin signaling and not necessarily by hyperglycemia itself. At P12, streptozotocin-treated mice with oxygen-induced retinopathy had significantly lower insulin plasma levels (Figure 4C). At P12, liver *Igf1* mRNA expression was decreased by more than 2-fold in the streptozotocin-treated oxygen-induced retinopathy group, before plasma glucose and IGF1 levels changed. By P17, the streptozotocin-treated oxygen-induced retinopathy group had developed hyperglycemia associated with a significant decrease of plasma IGF1 levels (Figure 5). The early downregulation of liver *Igf1* expression without affecting plasma glucose levels suggested a hyperglycemia-independent suppression of liver *Igf1* mRNA expression in this model, likely due to the hypoinsulinemia resulting in decreased insulin signaling in the liver. Our data are in line with those of previous reports showing a decrease in IGF1 expression in streptozotocin-treated rats (27, 28). Ex vivo studies in hepatocytes show that insulin alone can increase *Igf1* expression in a dose-dependent manner (29).

Insulin has been shown to increase IGF1 expression experimentally, but in the cohort of the Neonatal Insulin Therapy in Europe Trial Early (NIRTURE) (8) and in a study investigating intensive insulin treatment in critically ill children (30) insulin supplementation failed to show a relevant increase of IGF1 levels, with a decrease of IGF1 even seen in the latter study. It has been suggested that the lack of increase in IGF1 levels results from suppression of endogenous insulin production by exogenous insulin leading to decreased hepatic IGF1 expression. In line with this hypothesis, van Dijk et al. (31) showed that serum IGF1 levels are higher in patients with type 1 diabetes treated with insulin through the intraperitoneal route versus the s.c. route. A large retrospective study with 372 infants identified insulin treatment as an independent risk factor for ROP after adjusting for other major risk factors (32), possibly due to suppression of endogenous insulin production leading to decreased hepatic IGF1 expression.

To assess whether IGF1 supplementation can promote revascularization and therefore decrease neovascularization in oxygen-induced retinopathy in the setting of disturbed insulin signaling, we injected recombinant IGF1 starting at P12 when revascularization of the avascular retina begins. IGF1 treatment improved revascularization and decreased the avascular area at P17 (*P* = 0.027). In accordance with these findings, neovascularization at P17 was also significantly decreased (*P* < 0.0001) (Figure 6). Interestingly, IGF1 treatment did not impact body weight and glucose levels compared with control treatment (data not shown). IGF1 has been shown to have proangiogenic properties (33, 34). Clinical and preclinical studies suggest an essential role of IGF1 in developmental angiogenesis (13, 15, 35–37). These observations are in agreement with our results showing decreased revascularization of the avascular retinal area in hyperglycemic/hypoinsulinemic versus normoglycemic oxygen-induced retinopathy mice with low IGF1 levels and an improvement of retinal revascularization with IGF1 supplementation leading to decreased retinopathy.

The link between oxygen-induced retinopathy and IGF1 supplementation was evaluated by Vanhaesebrouck et al. (38); they found that a single bolus injection at P4 led to increased body weight and less neovascularization at P17. We analyzed circulating recombinant IGF1 levels after s.c. injection. The IGF1 serum level increased 250% compared with that in controls 1 hour after injection, but at 4 hours IGF1 was only 6.7% above that of controls (Supplemental Figure 1; supplemental material available online with this article; https://doi.org/10.1172/jci.insight.140363DS1). So, in our study, at P12, when revascularization after oxygen exposure begins, we initiated twice daily IGF1 injections due to the short half-life of recombinant IGF1.

A multicenter phase II randomized controlled IGF1 treatment trial sponsored by Shire (now Takeda) has recently been published (39). Supplementation with continuous intravenous infusion of rhIGF1/rhIGFBP-3 did not affect the primary endpoint, the development of ROP. One possible explanation for the lack of effect on ROP given by the authors was that target oxygen saturation levels in most neonatal intensive care units were increased at the beginning of the study period. This change was based on 3 landmark studies, which suggested that higher oxygen saturation targets decreased mortality but increased ROP (40–42). The strong effect of high oxygen promoting ROP might override the possible suppressive effect of IGF1 supplementation.

Our study has limitations. Clinically we do not have direct data on insulin insensitivity. Correlating glucose intake with glucose levels as a surrogate of insulin insensitivity has several limitations. Glucose levels change very dynamically, and small differences in glucose measurement time points after glucose intake can cause large variations in readings. In addition, other events, such as stress or infection, can significantly change glucose homeostasis independent of insulin sensitivity. However, it is very difficult to obtain direct insulin insensitivity measurements or insulin, C-peptide, and IGFBP3 measurements, because only very limited blood sampling is allowed in preterm infants. Further, it is not possible to investigate insulin insensitivity related to gestational age in mice, as mice are born at term with a normal IGF1/insulin axis (though eye development is delayed). Therefore, we used streptozotocin to decrease insulin levels to create a model with decreased insulin signaling, modeling insulin insensitivity. Even though streptozotocin has a high selectivity to insulin-producing β islet cells in rodents, it is important to note that other off-target effects, such as increased oxidative stress and inflammation, to other tissue have been described (43). Further, it is important to note that subtle adverse effects from IGF1 supplementation could have been missed, as other organ systems were not assessed in our experimental animal study. However, obvious side effects were not noted, and pups treated with IGF1 had body weights comparable to those of PBS-injected controls.

A strength of the study is the longitudinal aspect of the clinical data. We were able to correlate different glucose values with IGF1 levels. Only the high mean glucose tertile group had a significant decrease in IGF1 at P28. This could help predict preterm infants who might benefit from IGF1 treatment.

The mouse study showed that low insulin signaling was associated with low IGF1 levels and associated with increased retinal neovascularization (ROP). We found that hyperglycemia/hypoinsulinemia decreased revascularization and that IGF1 treatment improved revascularization, which is potentially novel and adds to the knowledge of how IGF1 treatment might help prevent ROP. Finally, the IGF1 treatment phenotype is novel to our knowledge.

In summary, we found that mean plasma glucose levels >7.51 mmol/L and signs of insulin insensitivity during early postnatal life were associated with lower postnatal IGF1 levels and increased risk of ROP. These findings were replicated in a neonatal hyperglycemia oxygen-induced retinopathy mouse model, demonstrating that decreased insulin signaling suppressed liver production of *Igf1* and lowered serum IGF1 levels and increased retinal neovascularization. IGF1 supplementation improved physiologic retinal revascularization, leading to decreased pathological neovascularization. The data further support the use of IGF1 supplementation as a potential treatment to prevent ROP, especially in very preterm infants with hyperglycemia in the first week, when IGF1 levels are further compromised.

## Methods

### Clinical cohort.

Data were retrieved from 2 prospective longitudinal cohort studies. Donna Mega (NCT02760472), a randomized controlled trial, evaluating fatty acid supplementation, was conducted at the Queen Silvia Children’s Hospital (44). Infants with gestational age <28 weeks who were admitted to the neonatal intensive care unit from April 4, 2013 to September 22, 2015 were included. For the present study, a total of 77 subjects from Donna Mega were eligible for analysis. An observational clinical study was conducted between January 2005 and May 2007 at Skane University Hospital (12, 45). For the present study, a total of 40 subjects were eligible for analysis. The inclusion criteria included the following: signed informed consent from parents/guardians and subject age of <28 weeks of gestation at birth. The exclusion criteria included the following: detectable clinical gross malformation; known or suspected chromosomal abnormality, genetic disorder, or syndrome according to the investigator’s opinion; fish allergy or other severe allergy in the mother; clinically significant neuropathy, nephropathy, retinopathy at birth, or micro- or macrovascular disease requiring treatment according to the investigator’s opinion; any other condition or therapy that, in the investigator’s opinion, may pose a risk to the subject or interfere with the subject’s ability to be compliant with this protocol or interfere with interpretation of results; and bleeding disorders.

The presence of a diagnosis of ROP, bronchopulmonary dysplasia, and intraventricular hemorrhage was noted. ROP screening started at 5–6 weeks of postnatal age. Retinal examinations were performed through dilated pupils during the full postnatal course (biweekly to once a week depending on ROP severity) until the retina was fully vascularized (a mean of 7 screening exams per infant). ROP was classified according to the international classification (46): stage 1, demarcation line separates avascular from vascularized retina; stage 2, ridge arising in region of demarcation line; stage 3, extraretinal fibrovascular proliferation/neovascularization; stage 4, partial retinal detachment; stage 5, total retinal detachment. If there were any asymmetric findings for ROP the more severe eye determined the staging of the infant. For treatment of ROP the recommendations of the Early Treatment for Retinopathy of Prematurity Cooperative Group (47) were followed.

### Nutrition regimes, including parenteral glucose intake, in preterm infants.

Intravenous glucose intake (mg/kg/min) was calculated prospectively from registered volume (mL) and concentration (mg/mL) of all intravenous glucose solutions from birth up to P7. For the Lund study, this was collected from clinical record form and for the Donna Mega study all nutritional data were registered in the computer-aided nutritional calculational program Nutrium (https://www.nutrium.se/). We did not calculate the oral administration of donor maternal breast milk or formula, as the major sugar in these milks is lactose. The lactose concentration in human milk is about 170 mM. Once hydrolyzed in the gut, human milk would provide 170 mM glucose and 170 mM galactose. This would be about 30 mg/mL glucose, assuming complete hydrolysis, absorption, and transport to the systemic circulation. In adult studies, it is more likely that only 80% that reaches the systemic circulation. Therefore, we estimated the glucose intake from the parenteral nutrition only.

### Quantitative analyses of plasma glucose in preterm infants.

Blood sampling for plasma glucose was performed from an umbilical arterial catheter or a peripheral arterial line. Plasma glucose was analyzed locally at the respective neonatal intensive care unit using Siemens RAPIDPoint 500 in the Donna Mega cohort and ABL 735 (Radiometer) in the Lund cohort. In total, 3511 measurements of plasma glucose were recorded the first week (median, 29; range, 7–73). Hyperglycemia was defined as a blood glucose value above 8.3 mmol (19) and hyperglycemia needing intervention as a blood glucose value above 10 mmol (48).

### Blood sampling and quantitative IGF1 measurements in preterm infants.

The blood samples were obtained initially from umbilical or peripheral arterial catheters and thereafter by venous puncture. Serum samples were taken at birth and then weekly for the first 5 postnatal weeks. After centrifugation, serum samples were stored at –80°C until assayed. For IGF1 analysis, the samples were diluted 1:50 with sample buffer, and the IGF1 concentrations were analyzed using insulin-like binding protein-blocked radioimmunoassay (Mediagnost) in the same assay, as described previously (49). The intraassay coefficients of variation for IGF1 were 18%, 11%, and 7% at concentrations of 9, 33, and 179 μg/L respectively.

### Neonatal hyperglycemia oxygen-induced retinopathy mouse model.

The well-described oxygen-induced retinopathy model (50) was combined with streptozotocin injections to induce hyperglycemia/hypoinsulinemia by destroying insulin-producing pancreatic β cells. Briefly, C57BL/6 mouse pups with S129 nursing dams were exposed to 75% oxygen from P7 to P12 in an oxygen chamber and returned to room air from P12–P17. Streptozotocin or littermate control PBS injections were performed intraperitoneally daily from P2 to P12 (25 μg per gram body weight). Retinas were collected at P12 and P17. Vaso-obliteration (area of avascular retina) and neovascularization were quantified using a fully automated deep-learning segmentation (51).

### Mouse IGF1 treatment.

In the hyperglycemia oxygen-induced retinopathy model, pups were treated with recombinant IGF1 (4 μg per gram body weight; PeproTech, 100-11) or PBS s.c. twice daily from P12 to P16. Retinas were collected at P17 and quantified as described above.

### RNA analysis.

Livers were immediately removed after eye enucleation and washed in ice-cold RNase-free PBS to remove excessive blood. A small piece of liver (~1 mm^2^) was dissected from the left lateral lobe and immediately flash frozen. The liver was lysed using QIAzol lysis reagent and was subject to mechanical disruption using motor-driven pellet pestles. Chloroform was added to the homogenate, vortexed rigorously, incubated at room temperature for 5 minutes, and then centrifuged at 12,000*g* for 15 minutes at 4°C. RNA extraction from the clear upper separation was performed using the PureLink RNA Mini Kit (Invitrogen, 12183018A). The RNA was reverse transcribed using the iScript cDNA synthesis kit (Bio-Rad, 1708891). A SYBR-Green–based (bimake.com, B21203) real-time PCR was performed for quantification. Gene expression was calculated relative to β_2_ microglobulin (*B2m*). The RT qPCR primer pairs are provided in Supplemental Table 1.

### Mouse plasma collection, glucose measurement, and protein analysis.

Blood was collected from the retro-orbital plexus right after enucleation into EDTA-coated tubes to prevent coagulation. The collected blood was centrifuged for 20 minutes at 2000*g* within 30 minutes of collection. Supernatant was collected, diluted, and assayed for IGF1 and insulin using ELISA kits following the manufactures’ standard protocols (IGF1, Quantikine ELISA, R&D Systems, MG100 and DG100; insulin, Crystal Chem, 90080). Glucose was measured using a commercially available fingerstick glucose meter (Clarity Diagnostic, BG1000).

### Animals.

WT C57BL/6 mice from The Jackson Laboratory were used for all experiments. 129 S6 mice from Taconic were used as surrogate nursing dams. Male and female animals were included in this study.

### Statistics.

Statistical analysis was performed using GraphPad Prism 6, IBM SPSS statistics 20 for Microsoft Windows, and SAS 9.4.

For clinical data analysis, relationships between plasma glucose levels and gestational age, birth weight, birth weight standard deviation score, and glucose intake, respectively, were analyzed using plasma glucose values obtained from P1 to P7. The Spearman’s rank test was used for assessment of correlations between continuous variables. Partial correlation was used for correcting for gestational age at birth. Mantel-Haenszel χ^2^ test was used for testing frequencies on ordinal data. Infants were divided into tertiles according to their mean plasma glucose concentrations from P1 to P7. Multivariable regression models were validated according to common practices: the error terms were normally distributed, residuals exhibited homoscedasticity, error terms were independent, no or few outliers were observed, and no multicollinearity was found. Parameters used to describe models from the regression analyses were β, the slope of the specified independent variable in the model; 95% CI for β; *P* value, the significance of the specified independent variable in the model; and model R^2^, the goodness of fit of the model. *P* values below or equal 0.05 were considered significant.

For preclinical data analysis, a 2-tailed unpaired *t* test was used for comparison between 2 groups. *P* values below or equal 0.05 were considered significant.

### Study approval.

The clinical studies were approved by the Regional Ethical Review Boards in Lund and Gothenburg, Sweden, and carried out in accordance with the 1964 Declaration of Helsinki and its later amendments or comparable ethical standards. All animal studies adhered to the Association for Research in Vision and Ophthalmology Statement for the Use of Animals in Ophthalmic and Vision Research and were approved by the Boston Children’s Hospital IACUC. International guidelines for ROP classification and ROP treatment were followed (47, 52).

## Author contributions

WH, CL, and AH designed and performed the clinical research and collected and analyzed data. AW, IHP, and DL assisted with data collection and statistical analyses for the clinical research. BC, YT, ZF, RL, and LEHS designed and performed the preclinical research and collected and analyzed data. The manuscript was written by BC, CL, and WH, with input from AH and LEHS, and reviewed by all authors. Co–senior authors AH and LEHS supervised the project throughout.

## Supplementary Material

Supplemental data

ICMJE disclosure checklist

ICMJE disclosure forms

## Figures and Tables

**Table 1:**
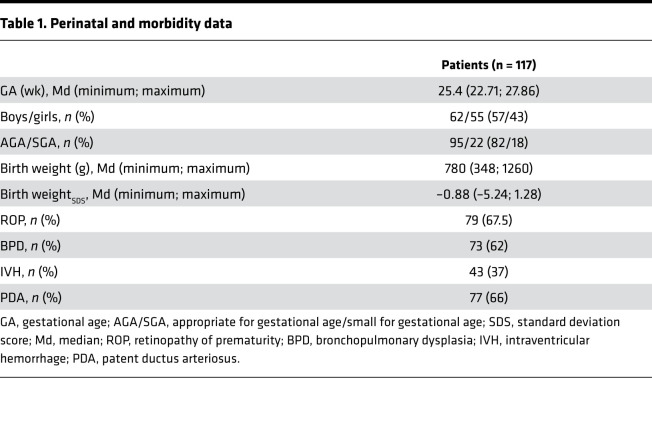
Perinatal and morbidity data

**Table 2:**
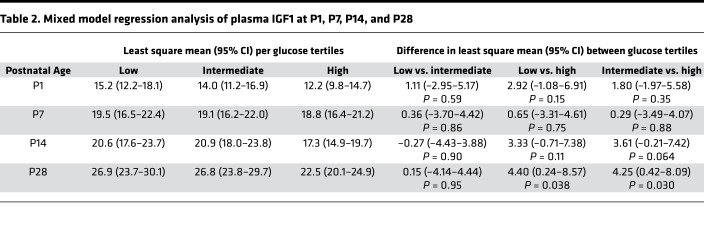
Mixed model regression analysis of plasma IGF1 at P1, P7, P14, and P28

## Version 1 10/02/2020

Electronic publication
