# Supplementary material for: IGF1, serum glucose, and retinopathy of prematurity in extremely preterm infants
Source: JCI Insight. 2020 Oct 2;5(19):e140363. doi: 10.1172/jci.insight.140363 (PMC7566718; doi:10.1172/jci.insight.140363)
Supplement: Supplemental data [file jciinsight-5-140363-s145.pdf]

CLINICAL STUDY PROTOCOL

A Randomised Intervention, Single-Center Study to Determine the Role of Fatty Acids in Serum in preventing Retinopathy of Prematurity

**Protocol Number:**	Omega 3-11-9
**Date:**	111121
**Sponsor and Principal Investigator:**	Ann Hellström M.D., Ph.D. [1]<br>Tel : +46 (0)31 3435774<br>Fax : +46 (0)31 3435771
**Principal investigator:**	Eva Engström M.D., Ph.D. [1]
**Scientific Advisers**:	Birgitta Strandvik, M.D., Ph.D. [1]<br>Lois Smith MD PhD [2]
**Clinic:**	[1]Göteborg University / Östra<br>The Queen Silvia Children's Hospital<br>[2]Harvard Medical School<br>Children's Hospital
**Producer of Drug:**	**SMOFlipid:** Fresenius-Kabi AB<br>Marknadsbolaget, 751 74 Uppsala<br>Phone: +46 18-64 49 00

This clinical study will be conducted, and essential study documentation archived, in compliance with requirements of the ICH Guidelines for Good Clinical Practice and Swedish laws

PROTOCOL SYNOPSIS

**Protocol Title:**	A Randomized, Intervention, Single-Center Study to Determine the Role of Fatty Acids in Serum and Breast Milk in preventing Retinopathy of Prematurity
**Protocol Number:**	Omega 3-0910
**Number of Subjects:**	45 subjects receiving Omega-3 supplementation and 45 subjects receiving conventional fatty acid supplementation.
**Subject Population:**	90 premature male/female infants, born before gestational age 28 weeks+ 0 days.
**Dosage:**	All infants on parenteral nutrition from birth and as long as clinically indicated are given fatty acid supplementation (Clinoleic). In addition, children randomized to Omega 3 supplementation receive SMOFlipid® with a quotient between Omega-6 and Omega 3 that is 2.5:1.
**Study Duration:**	From birth to 40 postmenstrual weeks
**Primary Study Objectives:**	A) The objective of this study is to determine how fatty acid (FA) levels in prematurely born children are affected by supplementation of “physiologic levels of long chain polyunsaturated fatty acids (LCPUFA) i.e. Omega-3 and 6<br><br>B) To determine whether these FA levels protect against development of retinopathy of prematurity (ROP)
**Secondary Study Objective:**	C) To determine whether these FA levels normalize growth (length, weight, head circumference) and/or<br><br>D) To determine whether these FA levels reduce the risk of lung, brain and gut morbidity
**Efficacy Endpoints:**	To compare in supplemented versus conventionally treated children<br><br>A) Serum and breast milk FA levels at days 1, 7, 14, 28 and in postmenstrual weeks 32, 36 and 40 and from breast milk FA levels day 7 and postmenstrual weeks 36 and 40.<br>B) Development of ROP<br>C) SDS score with regard to length, weight and head circumference growth.<br>D) Bronchopulmonary dysplasia, brain development on MRI examination and necrotizing enterocolitis

Safety Endpoints: Adverse events, clinical chemistry, retinal exam, physical examination, vital signs.

Table of Contents

1 INTRODUCTION 7

2 RATIONALE 8

3 STUDY OBJECTIVES 8

4 STUDY DESIGN 8

5 STUDY SUBJECTS 11

5.1 NUMBER OF SUBJECTS 11
5.2 INCLUSION CRITERIA 11
5.3 EXCLUSION CRITERIA 11

6 STUDY TREATMENT 12

6.1 INVESTIGATIONAL PRODUCT 12
6.2 STUDY DRUG ADMINISTRATION AND DOSING 12
6.3 BLINDING 13

7 STUDY CONDUCT 13

7.1 ETHICS AND REGULATORY CONSIDERATIONS 13
7.2 INDEPENDENT ETHICS COMMITTEE 13
7.3 INFORMED CONSENT/ASSENT FORM 14

8 STUDY EVALUATIONS 15

8.1 EFFICACY EVALUATIONS 15
ROP EXAMINATION 16
GROWTH 16
8.2 SAFETY EVALUATIONS 16
8.3 OTHER EVALUATIONS 17

9 SAFETY 17

9.1 RECORDING ADVERSE EVENTS 17
9.2 REPORTING SERIOUS ADVERSE EVENTS 19

10 STUDY MANAGEMENT 20

10.1 SUBJECT DISCONTINUATION 20
10.2 STUDY TERMINATION 21
10.3 DATA RECORDING 21
10.4 CASE REPORT FORMS 22
10.5 TRAINING 22
10.6 SOURCE DATA 23
10.7 QUALITY ASSURANCE 23
10.8 PROTOCOL AMENDMENTS 24
10.9 RETENTION OF STUDY RECORDS 24

11 DATA MANAGEMENT AND STATISTICAL METHODS 24

11.1 DETERMINATION OF SAMPLE SIZE 25
11.2 STUDY POPULATIONS 26

11.3 BACKGROUND AND DEMOGRAPHIC CHARACTERISTICS 26
11.4 ANALYSIS OF EFFICACY PARAMETERS 26
11.5 ANALYSIS OF SAFETY/TOLERABILITY 26
REFERENCES **28**
APPENDIX I **29**

Study Administrative Structure

**Sponsor and Principle Investigator:**	**Ann Hellström M.D., Ph.D.**<br>Göteborg University / Östra<br>The Queen Silvia Children's Hospital<br>SE – 416 85 GÖTEBORG<br>Tel : +46 (0)31 3435774<br>Fax : +46 (0)31 3435771<br>E-mail : ann.hellstrom@oft.gu.se
**Principal Investigator:**	**Eva Engström M.D., Ph.D.**<br>Göteborg University / Östra<br>The Queen Silvia Children's Hospital<br>SE – 416 85 GÖTEBORG<br>Tel: +46 (0)31 3434000<br>E-mail: eva.engstrom@vgregion.se
**Producer of Drug:**	**SMOFlipid®: Fresenius Kabi**<br>Anki Book<br>Tel: +4618644238<br>Mail: anki.book@fresenius-kabi.com
**Clinical Research Organization (CRO):**	Contact person: Carola Mosesson<br>**Pediatric Growth Research Center (GPGRC)**<br>Sahlgrenska University Hospital<br>The Queen Silvia Children's Hospital<br>SE- 416 85 Göteborg, Sweden<br>Tel:+46 709126127<br>Mail:carola.pfeiffer-mosesson@vgregion.se
**Laboratories:**	**Tillväxtlaboratoriet**,<br>Contact person: Irene Leonardsson<br>**Pediatric Growth Research Center (GPGRC)**<br>Sahlgrenska University Hospital<br>The Queen Silvia Children's Hospital<br>SE- 416 85 Göteborg, Sweden<br>Tel: +46 (0)31 3434793<br>Mail:irene.leonardsson@vgregion.se<br><br>**Laboratoriemedicin/Klinisk kemi**, Sahlgrenska Universitetssjukhuset, GÖTEBORG

List of Abbreviations and terms

AE	Adverse Event
AF	Amniotic Fluid
ALT	Alanine Aminotransferase
AST	Aspartate Aminotransferase
BUN	Blood Urea Nitrogen
CBC	Complete Blood Count
CRF	Case Report Form
CRO	Contract Research Organization
ECG	Electrocardiogram
GA	Gestational Age
GCP	Good Clinical Practice
GGT	Gamma Glutamyl Transferase
GMP	Good Manufacturing Practices
GW	Gestational Week
H	Hour
HC	Head Circumference
ICH	International Conference on Harmonisation
IV	Intravenous
LDH	Lactate Dehydrogenase
Min	Minute
MPA	Medical Products Agency of Sweden
MRI	Magnetic Resonance Imaging
NV	Neovascularisation
QA	Quality Assurance
RBC	Red Blood Cell
REG	Regionala Etikprövningsnämden i Göteborg (Ethics Committee)
ROP	Retinopathy of Prematurity
SAE	Serious Adverse Event
SD	Standard Deviation
SUSAR	Suspected Unexpected Serious Adverse Event
US	Ultrasound
VEGF	Vascular Endothelial Growth Factor
WHO	World Health Organization

1 INTRODUCTION

We hypothesize that when preterm children are deprived of their natural environment they lose important factors normally found *in utero*, such as proteins, essential fatty acids, growth factors and cytokines. Preterm birth is one of the main causes of long term morbidity of different organ systems. It is strongly associated with adverse neurological and cognitive outcome, growth retardation, cardiovascular and pulmonary morbidity, retinopathy of prematurity (ROP) and other ocular and visual disturbances, reflecting exposure to an abnormal environment during the last trimester.
In the eye, normal retinal vascularisation takes place between the fourth gestational month and term. A physiological hypoxia created by increased demands of the maturating retina increases the levels of vascular endothelial growth factor (VEGF) which promotes retinal angiogenesis. In preterm babies, delayed growth of vessels and hyperoxia induced vessel loss may be followed by uncontrolled neovascularisation causing retinal detachment and blindness.
Available treatment with laser and cryo-therapy is based on destruction of the peripheral retina and only reduces blindness by 25%.
In recent animal and human studies we have found that postnatal lack of the growth factor Insulin like growth factor I (IGF1) is associated with ROP and other prematurity related morbidity [1]. In addition, a major binding protein (IGFBP3) protects against oxygen induced vessel loss [2]. These are only two of many substances lost by interruption of placental supply.
The development of the central nervous system (including the retina) which consists of 60% fat is highly dependent on fatty acids for proper development especially during the third trimester when growth is intense. The role of lipids in this process in the regulation of angiogenesis is largely unknown. Human breast milk (HM) contains long chain polyunsaturated fatty acids (LCPUFAs), such as omega 3 (docosahexaeonic acid, DHA) and omega 6 (arachidonic acid, AA), which are the major LCFPUFA components in membranes of the brain and retina, and which are absent from many infant formulas. Breast-fed infants have higher levels of DHA than formula-fed infants and are reported to have better cognitive development [3] and visual acuity, although some studies found no difference between the two groups [4]. In a recent review article it was concluded that preterm infants have a high need for DHA and AA to allow rapid brain and body growth. The content of AA is fairly constant in HM averaging about 0.45% of total FA while DHA levels vary with mothers´ diet (0.1-3.8%). A AA to DHA ratio of approximately 1.5 with a DHA content of 0.4% has been recommended for preterm infants[4].
In rats, DHA content in the rod outer segment increases rapidly postnatally from 30 to 70% of total FA [5]. A precursor of DHA is found in the retinal vascular endothelium [6].
In a ROP mice model, we found that increasing the omega-3 PUFA levels by dietary (to the mothers) or genetic means decreased the avascular retinal area by increasing vessel re-growth after injury, thereby reducing the hypoxic stimulus for neovascularisation. Increased levels of omega-3 protected against pathological angiogenesis and the omega-3 derived mediators neuroprotectinD1, resolvinD1 and resolvinE1 also protected against neovascularisation. This protection was to a large part mediated through suppression of tumour necrosis factor α (TNFα) which was found in microglia adjacent to retinal vessels [7].
In a double-blind, randomized clinical trial of the effect of omega-3 fatty acids on the oxidative stress of preterm neonates fed through parenteral nutrition has been performed using this dose has been published (8).
The infants recruited in this study is similar to ours, the inclusion criteria were

(a) gestational age <32 weeks

(b) birth weight <1500 g
(c) admission in the ICU within 12 h after birth
(d) >80% of total energy would be taken in through PN, during the first 8 days of life
(e) the fat administration through PN would be started on the second day of life at the latest, with the maximum lipid dose being 3 g/kg/day
(f) anticipated duration of PN would be >7 days.

The source of fat was different for each one of the two groups studied. One group (A) was administered SMOFlipid®, a formulation containing medium-chain triacylglycerols (30%), lipids from soya bean oil (30%), olive oil (25%) and fish oil (15%), while one group (B) was administered the standard lipid formulation with lipids from soya bean oil (Intralipid 20%) Infants in both groups received the PN solutions for a trial period of at least 14 days, at the end of which each infant started oral feedings.
The mean day of life for the onset of enteral nutrition (breast milk) was the same for both groups (group A: 8.56±4.67 days, group B: 9.46±5.12 days, *P*-value: 0.702). The volume and rate of administration were similar between the two groups (group A: 31.78±17.19 ml, group B: 21.03±12.15 ml, *P*-value: 0.208, group A: 24.35±10.49 ml/kg, group B: 16.76±9.52 ml/kg, *P*-value: 0.136). In addition, enteral nutrition was <20% of total daily energy intake for the first 14 days of feeding.
Levels of vitamin E and total antioxidant potential TAP were significantly increased in group A, while in group B only vitamin A levels were significantly increased (*P*-value <0.05, data not shown) on the seventh day of the intervention. Body weight was not significantly altered in any group. Blood pressure and heart rate were significantly increased in both groups and at the same rate. Hematocrit values were less in both the groups, again with no difference between them. Hematocrit values were below the normal ranges. Platelet values increased in both groups (within normal ranges), while white blood cell count increased in group A, but still remained within normal ranges. Creatinine decreased significantly in group B (within normal ranges). Regarding vitamin levels, significant differences were observed between the two groups. Vitamin E levels increased only in group A, whereas vitamin A levels increased in both groups. However, there was no significant difference between the two groups regarding vitamin E values, at the end of the fourteenth day. On the other hand, vitamin A values were higher in group A than in group B at the end of the fourteenth day. This could not be completely attributed to the different interventions because there was a significant difference between vitamin A values at the beginning of PN. Moreover, the repeated measures analysis revealed no time × group effect for vitamin E and A levels. Interestingly, TAP was increased only in group A and the levels on the fourteenth day were significantly higher in group A than in group B. Moreover, significant time × group effect was noticed regarding TAP levels (*P*-value=0.001). Hence, the SMOFlipid® dose used for the present study has earlier been shown to be safe and have physiological antioxidant effects.
In addition, safety, tolerability, and efficacy of a novel lipid emulsion containing a mixture of soybean oil, medium-chain triglycerides, olive oil, and fish oil (SMOFlipid 20%) with reduced n-6 fatty acids (FA), increased monounsaturated and n-3 FA, and enriched in vitamin E were evaluated in premature infants compared with a soybean oil-based emulsion (9).
Sixty (30/30) premature neonates (age 3-7 days, gestational age ≤ 34 weeks, birth weights 1000-2500 g) received parenteral nutrition (PN) with either SMOFlipid 20% (study group) or a conventional lipid emulsion (Intralipid 20%, control group) for a minimum of 7 up to 14 days. Lipid supply started at 0.5 g · kg body weight(-1) · day(-1) on day 1 and increased stepwise (by 0.5 g) up to 2 g · kg body weight(-1) · day(-1) on days 4 to 14. Safety and efficacy parameters were assessed on days 0, 8, and 15 if PN was continued.

Adverse events, serum triglycerides, vital signs, local tolerance, and clinical laboratory did not show noticeable group differences, confirming the safety of study treatment. At study end, γ-glutamyl transferase was lower in the study versus the control group (107.8 ± 81.7 vs 188.8 ± 176.7 IU/L, $P < 0.05$). The relative increase in body weight (day 8 vs baseline) was 5.0% ± 6.5% versus 5.1% ± 6.6% (study vs control, not significant). In the study group, an increase in n-3 FA in red blood cell phospholipids and n-3:n-6 FA ratio was observed. Plasma α-tocopherol (study vs control) was increased versus baseline on day 8 (26.35 ± 10.03 vs 3.67 ± 8.06 µmol/L, $P < 0.05$) and at study termination (26.97 ± 18.32 vs 8.73 ± 11.41 µmol/L, $P < 0.05$).
In conclusion, parenteral infusion of SMOFlipid was safe and well tolerated and showed a potential beneficial influence on cholestasis, n-3 FA, and vitamin E status in premature infants requiring PN.
We now want to study whether supplementation of Omega-3 also protects against ROP in humans.

2 RATIONALE

Preterm Child
Most fatty acids, important for development and especially the omega 3 fatty acids for the brain development are transferred in the third trimester with means that in the premature infant this transport via the placenta is interrupted and the infant is dependent on the concentrations in breast milk which vary depending on the mother's diet and her stores. It has even been suggested that low Omega-3 would be a cause of premature delivery (Olsen SF 1992). Many countries have much higher levels of omega3 fatty acids in breast milk than found in Sweden and breast milk substitutions are generally now supplemented with the LCPUFA. Therefore the supplementation to be given cannot be seen to give any risks for the infant. On the contrary, several studies have shown that mother who eat equal to or less than twice fish a week during pregnancy give birth to infants with impaired development (Hibbeln, Landet 2007)
Low Omega-3 levels in premature infants between gestational ages of 23 and 40 weeks can be one reason for ROP development. Restoration of Omega-3 (DHA and EPA) to normal *in utero* levels may prevent ROP by allowing normal vessel growth and survival. An increase of Omega-3 levels bringing levels to within physiological range may prevent development of ROP (7).

Choice of study drug
Most diets today have high ratio of omega6/omega3 and there are several indicators that the ideal ratio for metabolism (i.e. prostaglandin synthesis) would be a ratio close to 2:1. The conventionally used Clinoleic has a ratio of 9:1, while SMOFlipid® has a ratio of 2.5:1.

3 STUDY OBJECTIVES

Primary objective

1) To determine how fatty acid (FA) levels in premature infants (born with a gestational age below 28 weeks (<28+0)) are affected by supplementation of "physiologic levels of long chain polyunsaturated fatty acids (LCPUFA) i.e. Omega-3 and 6.

2) To determine whether these FA levels protect against development of retinopathy of prematurity (ROP).

Secondary objective

1) To determine whether these FA levels normalize growth (length, weight, head circumference) and/or

2) To determine whether these FA levels reduce the risk of lung, brain and gut morbidity

4 STUDY DESIGN

The study is a Randomised Intervention, Single-Center Study to Determine the Role of Fatty Acids in Serum and Breast Milk in preventing Retinopathy of Prematurity
Subjects who meet all inclusion and none of the exclusion criteria will be enrolled into the study. Upon entry into the study, subjects will be randomized and given a unique subject number.
A randomised intervention study of 45+45 (number based on power analysis regarding up to date ROP frequency, see 5.1 and 11.1) infants without major malformations born with a gestational age less than 28 weeks + 0 days will be performed.
Randomization of the patients to either
1) Conventional fatty acid supplementation regime with Clinoleic (with Vitalipid infant and Soluvit supplementation) to the preterm infant
Or
2) SMOFlipid® (with Vitalipid infant and Soluvit supplementation), where the quotient of Omega 6:3 is 2.5:1 in order to mimic the physiologic relation of FAs in cord blood from birth, to the preterm infant.

Thus there is one group of infants (n=45) that will receive Omega-3 in the fatty acid supplementation. The time on parenteral nutrition and the amount of fatty acids given will be according to clinical routines. The randomization of the patients will be performed by the controller of the study Randomization will be in blocks with 10 children in each block.

Data collection
After we have received informed consent from the parents, blood samples 0.9 ml from the child will be taken according to present clinical practice at if possible from cord (2ml) and at days 0, 7, 14, 28 and in postmenstrual weeks 32, 36 and 40. Blood samples from the mothers for FA analyses will be taken after birth, day 7 and at gestational weeks 36 and 40. At the same time (except from day 1), breast milk samples are taken. Length, weight and head circumference are measured weekly.

Screening for ROP will be performed, at least once a week, according to clinical routines using a specific protocol.
We intend to analyze the content of phospholipids which can be done on small amounts of blood, is relatively insensitive to short term fluctuations in intake and mirror the composition of many membranes in the body. The analyses will be made using gas-liquid chromatography. The method has a coefficient of variability of 1-3% for the FAs concerned.

**Study Day ===>**	**0 (Birth)**	**Week 1**	**Week 2**	**28 GW**	**32 GW**	**36 GW**	**40 GW**	
**Examination / Sampling**								
**Informed Consent**	X							
**Maternal- & perinatal medical history**	X							
**Physical Exam/Apgar Score**	X							
**Vital signs (Heart rate, blood pressure, pO_2, breath frequency)**								
**Weight, length, HC**	X	X	X	X	X	X	X	
**Adverse Events**	X	X	X	X	X	X	X	
**Cranial/cerebral US**	X	X	X	X	X	X	X	
**Fatty acid sampling- serum (child)**			X					
**Fatty acid sampling-breast milk (mother)**	X	X	X	X	X	X	X	
**Chemistry and Hematology**		X	X	X	X	X	X	
**Study Enrollment**	X	X	X	X	X	X	X	
**Parenteral SMOFlipid®/ Clinoleic[1]**	X							
**MRI of the brain**	X	X	X					
**Clinical examination**							X	
**Neurological and cognitive examination**								
**Ophthalmological examination[2]**								

Table 1: Study events

[1] The time-period of parenteral nutrition will be according to clinical routines, varying from 1 day to maximum age 36 weeks PMA.

[2] Screening examinations for ROP will be performed weekly from five-six weeks after birth

5 STUDY SUBJECTS

5.1 Number of Subjects

For efficacy evaluations, 80 subjects need to be included for statistical considerations. However, to compensate for protocol violations and withdrawals up to 90 subjects will be included (see **11.1 De-termination of Sample Size and Statistical Methods** on page 26).
After the first 20 subjects have been treated and evaluated there will be a safety check to confirm that the safety profile is acceptable and that the assumption of a 50% reduction in ROP incidence is reasonable. A second safety check will be performed after the next 20 subjects.

5.2 Inclusion Criteria

Subjects must meet all the following inclusion criteria to be permitted into this study:

1. Signed informed consent from parents/guardians;
2. Subject must be below 28 weeks of gestation

5.3 Exclusion Criteria

Subjects presenting with any of the following will be excluded from the study:

1. Detectable clinical gross malformation;
2. Known or suspected chromosomal abnormality, genetic disorder, or syndrome, according to the investigator's opinion;
3. Fish allergy or other severe allergy in the mother
4. Clinically significant neuropathy, nephropathy, retinopathy, or other micro- or macrovascular disease requiring treatment, according to the investigator's opinion;
5. Any other condition or therapy that, in the investigator's opinion, may pose a risk to the subject or interfere with the subject's ability to be compliant with this protocol or interfere with interpretation of results.
6. Bleeding disorder.

6 STUDY TREATMENT

6.1 Investigational Product

Fresenius-Kabi AB has received marketing authorization for SMOFlipid® for parenteral nutrition.

Fresenius Kabi will supply SMOFlipid® for the study. The pharmacy at the study site will be responsible for storing and dispensing all supplies.

The formulation must be stored at +4 to +6.

6.2 Study Drug Administration and Dosing

Regarding fatty acid supplementation to preterm infants the today's regime at all neonatal intensive care units in Sweden is to prepare a mixture of Vitalipid infant and Soluvit with Clinoleic. In addition, according to today's clinical praxis and pharmacy regimes the preparation of Clinoleic, Vitalipid infant and Soluvit lasts for seven days if stored at +4 to +6 and prepared by the local pharmacy.

Preterm Child – Treated

All infants will receive parenteral and enteral nutrition according to clinical practice and routines.
The group of infants randomized to treatment with Omega-3 will receive SMOFlipid® (with Vitalipid infant and Soluvit supplementation) with a quotient between Omega 6 and Omega 3 that is 2.5:1. The time period for the parenteral nutrition will be according to the need of each infant and clinical practice.
There may be clinical situations when the infant do not tolerate any enteral feeding at all for a period leading to an increased dose of fat till at most 3 g/kg/ for a 1 kg infant over 24 hours. It is extremely rare in our neonatal intensive care unit that a WLBW infant is planned to receive more than 2 g fat/kg/24 h intravenously, but if it should happen the infant is always very thoroughly followed by clinical checkups, daily measurements of plasma triglycerides and blood gases.

Preterm Child - Conventional fatty acid supplementation

A preterm child that is randomized to conventional treatment receives Clinoleic (with Vitalipid infant and Soluvit supplementation) according to regular clinical practice and routines as described below.
For all preterm infants the parenteral nutrition with glucose, aminoacids and lipids is introduced within the first 24 hours of life. The intake of lipid emulsion (Clinoleic 20%) is gradually increased during the first days of life with an initial dose of 0.5 g/kg given over 24 hours to a maximum of 2 g/kg/24 h at day 3 to 5 of life. Consequently a preterm infant with a birth weight of 1 kg will receive a maximum of 10 ml lipid emulsion/24 h (2 g/kg). The initial dose of SMOFlipid® will be 0.5 – 1.0 g fat/kg body weight (bw)/day followed by a

successive increase by 0.5 – 1.0 g fat/kg bw/day up to 3.0 g fat/kg bw/day. We will not to exceed a daily dose of 3g fat/kg bw/day, corresponding to 15 mL SMOFlipid®/kg bw/day. The rate of infusion will not exceed 0.125 g fat/kg bw/hour.
This is, according to Brans et al, (Brans, Y.W.,Andrew, D.S., Carillo, D.W. et al. Tolerance of fat emulsions in very-low-birth-weight neonates. Am. J. Dis. Child. 1988;142:145-52) is within acceptable levels (i.e 2-3 g/kg/day) for WLBW infants. Enteral feeding with human breast milk is introduced from the first day of life. According to our clinical practice we start with 1-5 ml/kg every third hour depending of maturity and birth weight. The amount of breast milk is slowly and carefully gradually increased during the first 7 to 10 days of life until the infant is fully enteral fed. With increasing enteral amounts the parenteral dose of lipid emulsion is decreased. Some of the most immature infants may have problems to tolerate increased enteral feeding leading to a longer period of parenteral feeding in low doses. In conclusion, feeding WLBW infants according to clinical practice and routine are a regiment with *partial* parenteral nutrition combined with enteral nutrition.
If an infant do not tolerate any enteral feeding (for example gut problems as NEC or during any kind of surgery) the parenteral intake of lipid emulsion may be increased to a maximum of 3g/kg/24 h. The maximum time SMOFlipid® will be administered is to a postmenstrual age of 36 weeks, if the infant still need fatty acid supplementation after this age it will receive fatty acids according to clinical routines. This amount of lipid emulsions is very rare in this patient group and the plasma triglycerides will always be checked although we still are within acceptable levels.

6.3 Blinding

This is a randomised study with blinded ophthalmologic assessment of ROP stage. Retinal examination will be performed approximately once weekly starting at four to five weeks of age according to a standardized protocol and to clinical screening praxis. The assessing, evaluation of ROP stage 3 or more, independent from the study, pediatric ophthalmologists will be unaware which infants are participating in the study.

7 STUDY CONDUCT

7.1 Ethics and Regulatory Considerations

This study will be conducted in accordance with current Good Clinical Practices (GCPs) and International Conference on Harmonization (ICH) recommendations, as well as all applicable local, state, and federal regulations and guidelines regarding the conduct of clinical trials.

7.2 Independent Ethics Committee

The protocol, informed consent form, and other written subject information must be submitted to the “Regionala Etikprövningsnämnden i Göteborg” (REG) and their written unconditional approval must be obtained prior to commencement of the study.

Verification of unconditional approval from REG of the protocol and the approved informed consent form will be forwarded to Kabi Fresenius, the manufacturer of the study medication, prior to shipment of study medication supplies to the site.

7.3 Informed Consent/Assent Form

Informed consent by the parents/guardians for each subject will be obtained before initiating any study procedures. Both parents/guardians will have to sign the informed consent. Informed consent should be obtained as soon as possible after birth and no later than 24 hours after birth. One copy of the signed and personally dated informed consent must be given to each parent/guardian and one signed and personally dated copy must be retained in the investigator's trial records. The "Declaration of Helsinki" recommends that consent be obtained from each potential subject or parents/guardians in biomedical research trials after the physician has explained to the individual the purpose, methods, anticipated benefits, and potential hazards of the trial and discomfort it may entail.

Potential subject's parents/guardians should also be informed of their right not to participate or to withdraw from the study at any time. If the individual is in a dependent relationship to the physician or gives consent under duress, an independent physician should obtain the informed consent. If the individual is legally incompetent (i.e., a minor or mentally incompetent), informed consent must be obtained from the parent, legal guardian, or legal representative in accordance with the law in Sweden. See Appendix No. III for the "Declaration of Helsinki".

If a protocol amendment substantially alters the study design or there is an increase of a potential risk to the subject:

- the informed consent form or subject information sheet must be revised and submitted to the MPA and REG for review and approval; and
- the approved revised form must be signed by parents to subject currently enrolled in the study; or
- the new form must be used to obtain consent from new parents prior to enrollment into the study.

7.4 Subject Data Protection

The Investigator is responsible for keeping a list of all subjects (who have been allocated subject numbers) including subject numbers, full names (of parent and child, if applicable) and parent addresses.

The parents/guardians should also be informed in writing and agree to the possibility of audits and /or monitoring by authorized representatives of the Sponsor, the manufacturer of the study drug, and/or regulatory authorities in which case a review of those parts of the laboratory records relevant to the study may be required.

The parents/guardians should be informed in writing and agree to that the results will be stored and analyzed in a computer, maintaining confidentiality in accordance with Swedish data laws and “Personuppgiftslagen” (PUL SFS 1998:204, SFS 1998:1191)

7.5 Biobank

Samples taken in this study will be stored in a biobank according to the Swedish biobank law ((SFS 2002:297). The biobank is registred at the national board of health and welfare in Sweden (Socialstyrelsen). Samples will be thrown away when the study is finished. No further analyses other than those specified in this protocol will be performed on the samples. The parents will be informed about this.

8 STUDY EVALUATIONS

8.1 Efficacy evaluations

All values obtained from efficacy evaluations should be recorded onto the case record form (CRF). Serum samples will be taken simultaneously with clinical blood sampling.

Fatty acid analyses
All laboratory values for clinical chemistry will be obtained from Klinisk kemi, Sahlgrenska Universitetssjukhuset, the accredited central laboratory at Sahlgrenska / Östra hospital, SWEDAC accrediation number 1240 according to their routines.

The analyses of fatty acids will be performed by Professor Birgitta Strandvik's laboratory at the Göteborg University /SU Östra.
Total lipids of serum will be extracted according to Folch et al. [10]. Serum phospholipids will be fractionated on a single SEP-PAK aminopropyl cartridge (Waters Corp., Massachusetts, USA) and eluated with methanol after washing with chloroform:isopropanol 2:1 and 2% HAc in ether. The fraction of lipids will be transmethylated in methanolic-HCL-3N at 80°C over 4 hours. The FA methyl esters will be separated by capillary gas-liquid chromatography in a Hewlett-Packard 6890 gas chromatograph equipped with a 30 m x 0.25 mm SP-2380 column; film thickness 20 µm. Helium at 1.4 ml/min will be used as carrier gas. The injector and detector temperatures will be 250°C. The column oven temperature is programmed from 60°C to 230°C at a heating rate of 8°C/min up to 155°C, 1.5°C/min up to 180°C and thereafter 6°C/min up to 230°C, were it is ran for 10 min. The separation will be recorded with HP GC Chem Station software (HP GC, Wilmington, DE). Heneicosanoic acid (21:0) will be used as internal standard and the FA methyl esters identified by comparison with retention times of pure reference substances (Sigma Aldrich Sweden AB, Stockholm, Sweden). The method has been described earlier (ref Peng YM, Zhang TY, Wang Q, Zetterström R, Strandvik B. Fatty acid composition in breast milk and serum phospholipids of healthy term Chinese infants during first 6 weeks of life. Acta Paediatr 2007;96: 1640-5).

ROP examination
The retinal examination will be performed once weekly starting at five to six weeks of age according to a standardized protocol and to clinical screening praxis. The ophthalmologic assessment will be performed with strict criteria according to general Swedish Guidelines issued by the Swedish Ophthalmological Society: The Guidelines are available at following link: www.swedeye.org/SOTA/rop/SOTA-ROP_2006.pdf.

Growth
Length, weight and head circumference will be registered weekly from birth until 40 weeks postmenstrual age and at 2.5 and 6 years.
Neurologic development
Cranial ultrasound will be performed according to clinical praxis. MRI of the brain will be performed at 40 weeks PMA.

At 2.5 years a clinical examination including neurologic evaluation (neurologist) cognitive evaluation with Bailey-test (psychologist) and ophthalmologic examination will be performed.

At 6 years a clinical examination including neurologic evaluation (neurologist) cognitive evaluation with WPPSI alt WISC, short visuo-motor test and a behavioral test and extensive ophthalmologic examination, including visual perception and morphologic and functional examination of the retina, will be performed.

8.2 Safety Evaluations

All values obtained from safety evaluations should be recorded onto the CRF. Safety evaluations consist of the following:

- **AE reporting**: include SAEs and AEs recorded starting from receiving informed consent until the final study examination / sampling. AE reporting is further outlined in section 9, Safety.
- **Physical examination:** A complete physical examination will be performed in all subjects at birth according to standard clinical routines.
- **Vital signs:** Heart rate, blood pressure, pO_2 and breath frequency will be monitored from birth throughout the study. Subjects are connected with continuous surveillance of heart beat, breath frequency and pO_2. An alarm will notify the personnel and if any true divergences have occurred, these will be recorded in the patient record and in the CRF. Children with artery catheter are always under continuous intra artery blood pressure control with alarm for any irregular episodes.
- **Retinal exam**: Retinal exam will be performed according to clinical screening protocol. Any deviations from normal will be described in the CRF.
- **Cranial/Cerebral ultrasound** will be performed in all subjects by a pediatric radiologist as clinical routine. Any deviations from normal will be described in the CRF.
- **Magnetic resonance imaging of the brain** will be performed in all subjects by a pediatric radiologist as clinical routine. Any deviations from normal will be described in the CRF.
- **Laboratory evaluations**: Laboratory assessments will be performed according to standard clinical routines. Blood samples for fatty acid analyses; 0.2 ml from the child will be taken according to present clinical practice at days 1, 7 and 14 and in postmenstrual weeks 28, 32, 36 and 40. At the same time (except from day 1), breast milk samples are taken and length, weight and head circumference are measured.

Blood samples from the mothers for FA analyses will be taken after birth, day 7 and at gestational weeks 36 and 40.
Analyses of triglycerides cannot be taken more frequently then described in the protocol as the small circulating blood volume of these very preterm infants will not allow more sampling than what is already specified in the protocol.

It is extremely rare in our neonatal intensive care unit that a WLBW infant is planned to receive more than 2 g fat/kg/24 h intravenously, but if it should happen the infant is always very thoroughly followed by clinical checkups with daily measurements of plasma triglycerides and blood gases (as a safety control of metabolic state).

Laboratory evaluations consist of:

Hematology: Hemoglobin, hematocrit and platelets.

Chemistry: Sodium, potassium, calcium, uric acid, creatinine, alkaline phosphatase, albumin, total bilirubin, AST, ALT.

Plasma glucose: Blood for measurement of p-glucose is obtained from routine daily blood gas measurements and will be recorded in the CRF throughout the study.

8.3 Other Evaluations

- **Maternal and perinatal history:** Maternal and perinatal history will be recorded as soon as possible after birth after questioning the subject´s parents and/or recorded from the mother's patient record for the pregnancy ("Förlossningsjournaler" FHV-1 and FHV-2).

- **Weight, Length and Head Circumference (HC):** Weight, length and HC of the subject will be recorded once weekly from birth to 40 postmenstrual weeks and at 2.5 and 6 years.

9 SAFETY

9.1 Recording Adverse Events

Adverse events must be recorded starting from the time of informed consent until the final study examination / sampling day. Any medical condition present at the initial study day (birth day), which remains unchanged or improves, should not be recorded as an adverse event at subsequent examination / sampling days. However, if there is **deterioration** of a medical condition that was present at the initial study day (birth, day 0), this should be considered a **new** adverse event and reported. This information is collected by examining the subject. Ongoing adverse events at the final study day should be followed until the event is resolved or remains stable.

Clinically significant changes (abnormalities), in the judgment of the investigator, in physical examination from the baseline exam will be recorded as an adverse event.

The following information must be collected and recorded for each AE:

- AE term (diagnosis)
- Start date of AE
- Stop date of AE
- Severity of AE (mild, moderate, or severe)
- Seriousness of AE (serious or non-serious)
- Action taken regarding study medication (none, study medication dose reduced, study medication interrupted, or study medication stopped);

- Action taken regarding AE
- AE outcome (resolved, ongoing, death, or lost to follow-up)
- AE causality (not related, possibly related, related)

The investigator should determine study drug relationship for each adverse event. AEs occurring prior to administration of the study drug will be considered as not related to the study drug. The Investigator will be using the following explanations for assessment of causality:

Not related

- The event is clearly related to other factors such as the subject's clinical state, therapeutic interventions, or concomitant drugs administered to the subject.

Possibly Related

- The event follows a reasonable temporal sequence from the time of drug administration,
- And/or follows a known response pattern to the trial drug,
- **But** could have been produced by other factors such as the subject's clinical state, therapeutic interventions, or concomitant drugs administered to the subject.

Related

- The event follows a reasonable temporal sequence from the time of drug administration,
- **And** follows a known response pattern to the study drug,
- **And** cannot be reasonably explained by other factors such as the subject’s clinical state, therapeutic interventions, or concomitant drugs administered to the subject,
- **And** either occur immediately following trial drug administration, **or** improves on stopping the drug, **or** reappears on repeat exposure, **or** there is a positive reaction at the application site.

Severity of an adverse event is defined as a qualitative assessment of the degree of intensity of an adverse event as is determined by the investigator or reported to him/her by the subject.

The assessment of severity is made irrespective of drug relationship or seriousness of the experience and should be evaluated according to the following scale:

1. Mild– The adverse event is easily tolerated.

2. Moderate– The adverse event is not easily tolerated.

3. Severe– The adverse event is incapacitating and the subject is unable to function.

It is up to the discretion of the Investigator, in the event of an AE or SAE, to temporarily discontinue study medication or to discontinue the subject from the study. The end of study examinations / samplings must be performed if the subject is discontinued from the study.

9.2 Reporting Serious Adverse Events

A serious adverse event (SAE) is defined as any adverse drug experience occurring at any dose that results in any of the following outcomes:

- Results in death,
- Is life-threatening (NOTE: The term "life-threatening" in the definition of "serious" refers to an event in which the subject was at risk of death at the time of the event; it does not refer to an event which hypothetically might have caused death if it was more severe),
- Results in inpatient hospitalization or prolongation of existing hospitalization,
- Results in a persistent or significant disability/incapacity, or
- Results in a congenital anomaly/birth defect.

Important medical events that may not result in death, be life-threatening, or require hospitalization may be considered a serious adverse drug experience when, based upon appropriate medical judgment, the event may jeopardize the subject and may require medical or surgical intervention to prevent one of the outcomes listed in this definition. Examples of such events include allergic bronchospasm requiring intensive treatment in an emergency room or at home, blood dyscrasias or convulsions that do not result in an inpatient hospitalization, or the development of drug dependency or drug abuse.

Any SAE occurring during the study (from receiving informed consent until end of study) or within 30 days after study completion will be reported to the Sponsor within 24 hours of knowledge of the event.

Initial SAE reporting can be done by telephone with written reports to follow by fax within 24 hours.

Sponsor Safety Contact Information

Contact: Ann Hellström
Title: MD Professor
Address: The Queen Silvia Children's Hospital
Göteborg University/Östra
SE – 416 85 GÖTEBORG
24 hour line: +46 (0)768 979196
Office: +46 (0)31 3435774
Fax: +46 (0)31 3435771

All SAEs must be followed until resolution (subject has returned to baseline status of health), or until stabilization (the investigator does not expect any further improvement or worsening of the reported event).

Some events may require immediate reporting to the appropriate regulatory authorities. The Sponsor is responsible for regulatory reporting. Events that are life-threatening, result in a subject's death, or are considered related to the use of the study drug and unexpected according to the current approved product information for the investigational product (SUSARs) must be reported to the appropriate regulatory authorities by fax or telephone within 7 days. A written report must follow within 15 days after the initial notification. All other SAEs that are considered related to the use of study drug and are not unexpected (per the current approved product information) will be reported according to ICH guideline, E2F-Note for guidance on development safety update reports, to the authorities once a year.

The Sponsor is responsible for notifying the MPA and REG of all SUSARs that occur in the study. The Sponsor has an agreement with Pharma Consulting Group in Uppsala AB (PCG), Uppsala Science Park, Dag Hammarskjölds Väg 40 C, 751 83 Uppsala. Any SAE occurring in the study will be reported to PCG who will determine if the SAE is a SUSAR or not. PCG will classify the SAE as a SAE or a SUSAR and report the event to the Eudravigilance database, coded according to MedDRA.

The Sponsor will provide data related to any AE deemed to be related or possibly related to SMOFlipid® administration to Fresenus Kabi AB within 7 days. All SAEs occurring in the study should be reported to Fresenius Kabi AB within 24 hours after the Sponsor receive knowledge of the event. The SAE Report Form should be completed according to the guidelines and faxed to Fresenius Kabi AB.

Safety Contact Information for Fresenius Kabi AB:

Contact: Fresenius Kabi AB
Address: Marknadsbolaget, 751 74 Uppsala
Fax: +46 018-64 49 20

10 STUDY MANAGEMENT

10.1 Subject Discontinuation

A discontinuation occurs when an enrolled subject ceases participation in the study, regardless of the circumstances, prior to completion of the protocol.

The investigator has the right to remove a subject at any time if it is in the best medical interest of the subject.

Subjects will be discontinued from treatment prior to completion for any of the following reasons:

- Consent withdrawn / the parents/guardians wish to discontinue study treatment
- AE (clinical events or laboratory values) that contraindicate continuing the study.
- Best interest of the patient, as judged by the Investigator
- Protocol violation

- Administrative Decision
- Other, by Investigator specified reason

Subjects withdrawn from the study for a SAE should be followed until the SAE has resolved. Appropriate supportive and/or definitive therapy should be administered as required.

The investigator must determine the primary reason for discontinuation. The reason for a subject discontinuing from the study will be recorded on the case report form. Withdrawal due to an adverse event should be distinguished from withdrawal due to other reasons, according to the definition of an adverse event noted earlier. A discontinuation must be reported immediately to the Kabi Fresenius AB safety monitor or his/her designated representative if it is due to an adverse event. The **End of Study** examination / sampling must be performed at the time of the study discontinuation. The investigator will record the reason for study discontinuation, provide or arrange for the appropriate follow-up (if required) for such subject, and document the course of the subject's condition.

10.2 Study Termination

Termination of the study before all subjects have been enrolled can occur for any of the following reasons:

- the Swedish Authorities requested termination of the study;
- it was determined that the risk level associated with the experimental drug was significant and warranted termination of the study;
- the sponsor terminated the study for any reason, at any time, by written notice of intended termination;
- the principal investigator, REG or MPA terminated participation of that clinical site in the study by written notice;
- any other clause in the individual site Clinical Study Agreement was not met

10.3 Data Recording

Source documents are original documents, data, and records from which the subject's case report form (CRF) data are obtained. These may include but are not limited to hospital records, clinical and office charts, laboratory and pharmacy records, diaries, microfiches, radiographs, and correspondence. All original source documents supporting entries on CRFs must be maintained and be readily available.

The investigator will record all data with respect to the study in the subject's CRFs. This includes but is not limited to study procedures, laboratory data, safety-related data and drug accountability.

The investigator will sign and date the indicated places on the CRFs. These signatures will signify that the investigator inspected or reviewed the data on the CRF and on the data queries and that he/she agrees with the content.

All corrections on a CRF and on source documents must be made in a way that does not obscure the original entry. The correct data must be inserted, dated and initialed by study center personnel. If the reason for the change is not obvious, an explanation should be provided.

10.4 Case Report Forms

The Investigator will complete Case Report Forms for all subjects. These are to be completed in English. If a test/assessment is not done and will not be available, indicate this by writing "N/D" (Not Done) in the respective answer field in the CRF. If the question is irrelevant (e.g. is not applicable) indicate this by writing "N/A" (Not Applicable) in the respective answer field.

Corrections of data can only be made by crossing out the incorrect data and writing the correct data next to those crossed out (e.g. ~~352~~ 325). Erasure by any method is not allowed. Any changes in the CRF by the Investigator or his/her delegate must be signed with initials, dated and explained (if necessary). If corrections are made by the Investigator's authorised staff after the date of the Investigator's signature on the CRF, the CRF must be signed and dated again by the Investigator. Corrections necessary after the CRFs have been removed from the Investigator's site must be documented on a Data Clarification Form (DCF).

Original source documents, case report forms, and other study documentation will be maintained at the study site as specified.

Completed original Case Report Forms are the property of the Sponsor.

10.5 Training

The Investigator will ensure that appropriate training relevant to the study is given to the medical, nursing and other staff involved. Any information of relevance to the performance of this study is to be forwarded to the co-Investigator and other staff involved.

All investigators signing the protocol and key personnel should provide signed and dated Curriculum Vitae (CV) originals to be filed by the Sponsor. The CV should include name, title, occupation, education, research experience and present and former positions. A staff signature list including delegation responsibilities is required and will be continuously up-dated.

10.6 Source Data

Source data will be collected in the "Source Data File". The Source Data File should contain raw data from examinations/samplings/laboratory results required to verify the data entered into each patient's CRF.

The following data will be recorded directly on the CRFs and will be considered source data: Gender, age, length, weight, HC, medical history, physical examination and retinal scan.

The hospital records should clearly indicate at least:

- that the patient participated in the study (by patient identification and study identification)
- when the written informed consent was obtained,
- all examinations/samplings/laboratory results of importance for the patient's clinical care and
- serious adverse events.

10.7 Quality Assurance

The data will be entered into a database, where internal review and programmed computer checks will be used to identify selected protocol violations and data errors. If necessary, requests for clarifications or corrections will be sent to the investigator.

The investigator agrees to monitoring of the study by a Sponsor representative and that Regulatory Authorities will have the right from time to time both during and after the course of this trial to inspect the study and review pertinent medical records relating to this clinical trial.

Before, during and after the study, the monitor will have regular contacts with the clinic including visits to confirm that facilities remain acceptable, that the investigational team is adhering to the protocol, that data are being accurately recorded in the CRF and to provide information and support to the Investigator. Monitoring of the study will be carried out by Department of Clinic Trials, Institute of Clinical Science, The Queen Silvia Children´s Hospital Gothenburg according to their Standard Operating Procedures (SOP)

A statement will be obtained from each subject's parents/guardians participating in the trial permitting the release of the subject's medical records as necessary for monitoring or inspection by authorized personnel for the Sponsor and Regulatory Authorities.

The investigator is responsible for maintaining a comprehensive and centralized filing system of all study-related (essential) documentation, suitable for inspection at any time by representatives of the Sponsor and Regulatory Authorities.

10.8 Protocol Amendments

All revisions to the protocol should be reviewed and approved by the Sponsor and Insmed prior to submission of the amendment to the regulatory authorities. If the revision is an Administrative Change, the investigator should submit it to the REG for their information. If the revision is an Amendment to the protocol, the Investigator must sign it to verify he/she has read and understands the change. The investigator must submit the Amendment to the REG for review and approval prior to implementation.

If an amendment substantially alters the study design or increases the potential risk to the subject:

- the informed consent form or subject information sheet must be revised and submitted to the REG for review and approval; and
- the approved revised form must signed by both parents/guardians currently enrolled in the study; or
- the new form must be used to obtain consent from new parents/guardians prior to their enrollment into the study.

10.9 Retention of Study Records

ICH-GCP guidelines require the medical records and notes, etc., should be clearly marked and permit easy identification of participation by an individual in the trial.

The files should be archived by the investigator at least 10 years after the study is finished.

The investigator is to record all data with respect to protocol procedures, drug administration, laboratory data, safety data, and efficacy data on the CRFs. Essential documents should be retained until at least 2 years after the last approval of a marketing application in an ICH region and until there are no pending or contemplated marketing applications in an ICH region or at least 2 years have elapsed since the formal discontinuation of clinical development of the investigational product. The investigator should store the study records in a secure location.

11 DATA MANAGEMENT AND STATISTICAL METHODS

The investigator will record all data with respect to the study in the subject's CRFs. This includes, but is not limited to, study procedures, laboratory data and safety-related data.

All corrections on a CRF and on source documents must be made in a way that does not obscure the original entry. The correct data must be inserted, dated and initialed by study center personnel. If the change is not obvious, an explanation should be provided.

Completed CRFs for this study will be forwarded to the sponsor for editing, construction of a quality-assured database, and analysis of the data.

11.1 Determination of Sample Size and Statistical Methods

Primary endpoint

The primary endpoint is ROP classified as 0,1,2,3,3+ which is the expected possible range of ROP stages that will be encountered in the study based on analysis of infants screen for ROP in Gothenburg through the years 2004 to 2008. This is an ordinal scale with higher numbers indicating a more severe outcome. The proportion of children with each outcome based on register data is given in table below. In this table an anticipated treatment effect is also indicated by presenting expected outcome in the treated group.

**ROP**	**0**	**1**	**2**	**3**	**3+**	Total
Control (%)	26	15	24	18	17	100
Active (%)	49	19	19	9	4	100

Percentage distribution of children with different ROP-outcomes. Control group based on register data and active group based on expected treatment effect.

The estimated treatment effect is based on the following assumptions.

- 25 % of the children will not have any benefit from the treatment.
- 25% will have their ROP reduced one step (e.g. from 2 to 1)
- 50% will have their ROP reduced two steps. (e.g. from 3+ to 2)

The null hypothesis to test is if the distribution (*F*) of ROP (*X*) is the same in both treatment groups.

$$H_0 : F_C(X) = F_A(X)$$
$$H_1 : F_C(X) \neq F_A(X)$$

This hypothesis will be tested with the Mann-Whitney test.

In order to have 80% power at a significance level of 5% a sample of 40 subjects per group is needed (80 subjects in total).

Secondary endpoints

Important secondary endpoints are:

- Body weight
- Length
- Head circumference

These will be modelled by a linear mixed effects model. Subject will be used as a random factor, and *GA* and *treatment* will be used as fixed factors. The hypothesis of interest is if there is a significant difference in the slope, i.e. that the growth rate is different in the two groups.

11.2 Study Populations

All available subjects will be used in the data summaries and the listings of subject data. If a subject is regarded as non-evaluable this subject may be listed separately and not included in the summary statistics.

11.3 Background and Demographic Characteristics

The background and demographic variables will be displayed in per subject listings. The quantitative variables will also be summarized by means, standard deviations and medians. The qualitative variables will be displayed in frequency tables.

11.4 Analysis of Efficacy Parameters

Fatty acid analysis
Serum concentrations of fatty acids will be reported for the child and mother and displayed in per subject listings and graphs. The efficacy parameters will be displayed in per subject listings and summarized by means, standard deviations and median values.
ROP evaluation
The ophthalmologic assessment will be performed with strict criteria according to general Swedish Guidelines issued by the Swedish Ophthalmological Society: The Guidelines are available at following link: www.swedeye.org/SOTA/rop/SOTA-ROP_2006.pdf.
The evaluation of ROP stage 3 or more will be assessed by retinal examination by a trained ophthalmologist and will be performed in a blinded fashion i.e. the ophthalmologist will be unaware of whether or not the infant is participating in the clinical study.

11.5 Analysis of Safety/Tolerability

Physical examination, MRI and the results of retinal examination will be displayed in per subject listing.

The vital signs and laboratory measurements will be listed per subject and illustrated graphically as levels per time-point (as per Table 1, page 11) by each subject. The measurements will also be summarized by descriptive statistics.

Adverse events will be displayed in per subject listings.

--- ❖ ---

REFERENCES

1. Hellstrom, A., et al., Postnatal serum insulin-like growth factor I deficiency is associated with retinopathy of prematurity and other complications of premature birth. Pediatrics, 2003. **112**(5): p. 1016-20.
2. Lofqvist, C., et al., IGFBP3 suppresses retinopathy through suppression of oxygen-induced vessel loss and promotion of vascular regrowth. Proc Natl Acad Sci U S A, 2007. **104**(25): p. 10589-94.
3. Anderson, J.W., B.M. Johnstone, and D.T. Remley, Breast-feeding and cognitive development: a meta-analysis. Am J Clin Nutr, 1999. **70**(4): p. 525-35.
4. Fleith, M. and M.T. Clandinin, Dietary PUFA for preterm and term infants: review of clinical studies. Crit Rev Food Sci Nutr, 2005. **45**(3): p. 205-29.
5. Suh, M., et al., Dietary 20:4n-6 and 22:6n-3 modulates the profile of long- and very-long-chain fatty acids, rhodopsin content, and kinetics in developing photoreceptor cells. Pediatr Res, 2000. **48**(4): p. 524-30.
6. SanGiovanni, J.P. and E.Y. Chew, The role of omega-3 long-chain polyunsaturated fatty acids in health and disease of the retina. Prog Retin Eye Res, 2005. **24**(1): p. 87-138.
7. Connor, K.M., et al., Increased dietary intake of omega-3-polyunsaturated fatty acids reduces pathological retinal angiogenesis. Nat Med, 2007. **13**(7): p. 868-73.
8. Skouroliakou et al. Eur J Clin Nutr. 2010 Sep;64(9):940-7. Epub 2010 Jun 16.
9. Tomsits E, Pataki M, Tölgyesi A, Fekete G, Rischak K, Szollár L. Safety and efficacy of a lipid emulsion containing a mixture of soybean oil, medium-chain triglycerides, olive oil, and fish oil: a randomised, double-blind clinical trial in premature infants requiring parenteral nutrition. J Pediatr Gastroenterol Nutr. 2010 Oct;51(4):514-21.
10. Folch J, L.M., Sloane-Stanley GH. 1957. A simple method for the isolation and purification of total lipids from animal tissues. J Biol Chem. **226:** 497-509.

APPENDIX I

WORLD MEDICAL ASSOCIATION DECLARATION OF HELSINKI

Ethical Principles for Medical Research Involving Human Subjects

Adopted by the 18th WMA General Assembly Helsinki, Finland, June 1964 and amended by the:
29th WMA General Assembly, Tokyo, Japan, October 1975
35th WMA General Assembly, Venice, Italy, October 1983
41st WMA General Assembly, Hong Kong, September 1989
48th WMA General Assembly, Somerset West, Republic of South Africa, October 1996
52nd WMA General Assembly, Edinburgh, Scotland, October 2000

A. INTRODUCTION

1. The World Medical Association has developed the Declaration of Helsinki as a statement of ethical principles to provide guidance to physicians and other participants in medical research involving human subjects. Medical research involving human subjects includes research on identifiable human material or identifiable data.
2. It is the duty of the physician to promote and safeguard the health of the people. The physician's knowledge and conscience are dedicated to the fulfillment of this duty.
3. The Declaration of Geneva of the World Medical Association binds the physician with the words, "The health of my subject will be my first consideration," and the International Code of Medical Ethics declares that, "A physician shall act only in the subject's interest when providing medical care which might have the effect of weakening the physical and mental condition of the subject."
4. Medical progress is based on research which ultimately must rest in part on experimentation involving human subjects.
5. In medical research on human subjects, considerations related to the well-being of the human subject should take precedence over the interests of science and society.
6. The primary purpose of medical research involving human subjects is to improve prophylactic, diagnostic and therapeutic procedures and the understanding of the aetiology and pathogenesis of disease. Even the best proven prophylactic, diagnostic, and therapeutic methods must continuously be challenged through research for their effectiveness, efficiency, accessibility and quality.
7. In current medical practice and in medical research, most prophylactic, diagnostic and therapeutic procedures involve risks and burdens.
8. Medical research is subject to ethical standards that promote respect for all human beings and protect their health and rights. Some research populations are vulnerable and need special protection. The particular needs of the economically and medically disadvantaged must be recognized. Special attention is also required for those who cannot give or refuse consent for themselves, for those who may be subject to giving consent under duress, for those who will not benefit personally from the research and for those for whom the research is combined with care.
9. Research Investigators should be aware of the ethical, legal and regulatory requirements for research on human subjects in their own countries as well as applicable international requirements. No national ethical, legal or regulatory

requirement should be allowed to reduce or eliminate any of the protections for human subjects set forth in this Declaration.

B. BASIC PRINCIPLES FOR ALL MEDICAL RESEARCH

10. It is the duty of the physician in medical research to protect the life, health, privacy, and dignity of the human subject.
11. Medical research involving human subjects must conform to generally accepted scientific principles, be based on a thorough knowledge of the scientific literature, other relevant sources of information, and on adequate laboratory and, where appropriate, animal experimentation.
12. Appropriate caution must be exercised in the conduct of research which may affect the environment, and the welfare of animals used for research must be respected.
13. The design and performance of each experimental procedure involving human subjects should be clearly formulated in an experimental protocol. This protocol should be submitted for consideration, comment, guidance, and where appropriate, approval to a specially appointed ethical review committee, which must be independent of the investigator, the sponsor or any other kind of undue influence. This independent committee should be in conformity with the laws and regulations of the country in which the research experiment is performed. The committee has the right to monitor ongoing trials. The researcher has the obligation to provide monitoring information to the committee, especially any serious adverse events. The researcher should also submit to the committee, for review, information regarding funding, sponsors, institutional affiliations, other potential conflicts of interest and incentives for subjects.
14. The research protocol should always contain a statement of the ethical considerations involved and should indicate that there is compliance with the principles enunciated in this Declaration.
15. Medical research involving human subjects should be conducted only by scientifically qualified persons and under the supervision of a clinically competent medical person. The responsibility for the human subject must always rest with a medically qualified person and never rest on the subject of the research, even though the subject has given consent.
16. Every medical research project involving human subjects should be preceded by careful assessment of predictable risks and burdens in comparison with foreseeable benefits to the subject or to others. This does not preclude the participation of healthy volunteers in medical research. The design of all studies should be publicly available.
17. Physicians should abstain from engaging in research projects involving human subjects unless they are confident that the risks involved have been adequately assessed and can be satisfactorily managed. Physicians should cease any investigation if the risks are found to outweigh the potential benefits or if there is conclusive proof of positive and beneficial results.
18. Medical research involving human subjects should only be conducted if the importance of the objective outweighs the inherent risks and burdens to the subject. This is especially important when the human subjects are healthy volunteers.
19. Medical research is only justified if there is a reasonable likelihood that the populations in which the research is carried out stand to benefit from the results of the research.

20. The subjects must be volunteers and informed participants in the research project.
21. The right of research subjects to safeguard their integrity must always be respected. Every precaution should be taken to respect the privacy of the subject, the confidentiality of the subject's information and to minimize the impact of the study on the subject's physical and mental integrity and on the personality of the subject.
22. In any research on human beings, each potential subject must be adequately informed of the aims, methods, sources of funding, any possible conflicts of interest, institutional affiliations of the researcher, the anticipated benefits and potential risks of the study and the discomfort it may entail. The subject should be informed of the right to abstain from participation in the study or to withdraw consent to participate at any time without reprisal. After ensuring that the subject has understood the information, the physician should then obtain the subject's freely-given informed consent, preferably in writing. If the consent cannot be obtained in writing, the non-written consent must be formally documented and witnessed.
23. When obtaining informed consent for the research project the physician should be particularly cautious if the subject is in a dependent relationship with the physician or may consent under duress. In that case the informed consent should be obtained by a well-informed physician who is not engaged in the investigation and who is completely independent of this relationship.
24. For a research subject who is legally incompetent, physically or mentally incapable of giving consent or is a legally incompetent minor, the investigator must obtain informed consent from the legally authorized representative in accordance with applicable law. These groups should not be included in research unless the research is necessary to promote the health of the population represented and this research cannot instead be performed on legally competent persons.
25. When a subject deemed legally incompetent, such as a minor child, is able to give assent to decisions about participation in research, the investigator must obtain that assent in addition to the consent of the legally authorized representative.
26. Research on individuals from whom it is not possible to obtain consent, including proxy or advance consent, should be done only if the physical/mental condition that prevents obtaining informed consent is a necessary characteristic of the research population. The specific reasons for involving research subjects with a condition that renders them unable to give informed consent should be stated in the experimental protocol for consideration and approval of the review committee. The protocol should state that consent to remain in the research should be obtained as soon as possible from the individual or a legally authorized surrogate.
27. Both authors and publishers have ethical obligations. In publication of the results of research, the investigators are obliged to preserve the accuracy of the results. Negative as well as positive results should be published or otherwise publicly available. Sources of funding, institutional affiliations and any possible conflicts of interest should be declared in the publication. Reports of experimentation not in accordance with the principles laid down in this Declaration should not be accepted for publication.

C. ADDITIONAL PRINCIPLES FOR MEDICAL RESEARCH COMBINED WITH MEDICAL CARE

28. The physician may combine medical research with medical care, only to the extent that the research is justified by its potential prophylactic, diagnostic or therapeutic value. When medical research is combined with medical care, additional standards apply to protect the subjects who are research subjects.
29. The benefits, risks, burdens and effectiveness of a new method should be tested against those of the best current prophylactic, diagnostic, and therapeutic methods. This does not exclude the use of conventional fatty acid supplementation, or no treatment, in studies where no proven prophylactic, diagnostic or therapeutic method exists.
30. At the conclusion of the study, every subject entered into the study should be assured of access to the best proven prophylactic, diagnostic and therapeutic methods identified by the study.
31. The physician should fully inform the subject which aspects of the care are related to the research. The refusal of a subject to participate in a study must never interfere with the subject-physician relationship.
32. In the treatment of a subject, where proven prophylactic, diagnostic and therapeutic methods do not exist or have been ineffective, the physician, with informed consent from the subject, must be free to use unproven or new prophylactic, diagnostic and therapeutic measures, if in the physician's judgment it offers hope of saving life, re-establishing health or alleviating suffering. Where possible, these measures should be made the object of research, designed to evaluate their safety and efficacy. In all cases, new information should be recorded and, where appropriate, published. The other relevant guidelines of this Declaration should be followed.

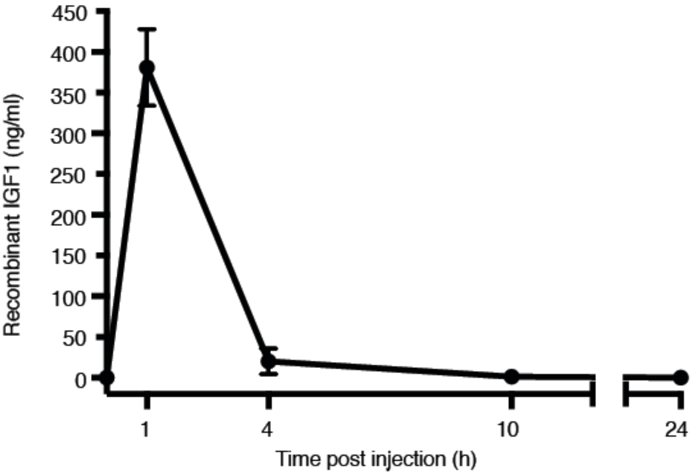

Figure S1. Post injection recombinant IGF1 plasma levels.
Recombinant IGF1 (4mg/KG) was injected subcutaneously in P17 wild type mice. Blood samples were collected 1, 4, 10 and 24h after injection for recombinant IGF1 measurements (n=2-3). *Error bars represent mean +/- SD.*

Supplemental Table 1. RT qPCR Primer Pairs.

Primer-Set	Sense	Antisense
Igf1	5’-GGCTCCAGCATTCGGAGGGC-3’	5’-CGCTGGGCACGGATAGAGCG-3’
Igfbp1	5’-ATCAGCCCATCCTGTGGAAC-3’	5’-TGCAGCTAATCTCTCTAGCACTT-3’
Igfbp3	5’-CCAGGAAACATCAGTGAGTCC-3’	5’-GGATGGAACTTGGAATCGGTCA-3’
B2m	5’-TTCTGGTGCTTGTCTCACTGA-3’	5’-CAGTATGTTCGGCTTCCCATTC-3’

CONSORT 2010 checklist of information to include when reporting a randomised trial*

Section/Topic	Item No	Checklist item	Reported on page No
**Title and abstract**			
	1a	Identification as a randomised trial in the title	1
	1b	Structured summary of trial design, methods, results, and conclusions (for specific guidance see CONSORT for abstracts)	5
**Introduction**			
Background and objectives	2a	Scientific background and explanation of rationale	7-8
	2b	Specific objectives or hypotheses	8
**Methods**			
Trial design	3a	Description of trial design (such as parallel, factorial) including allocation ratio	9-
	3b	Important changes to methods after trial commencement (such as eligibility criteria), with reasons	
Participants	4a	Eligibility criteria for participants	9
	4b	Settings and locations where the data were collected	9
Interventions	5	The interventions for each group with sufficient details to allow replication, including how and when they were actually administered	10
Outcomes	6a	Completely defined pre-specified primary and secondary outcome measures, including how and when they were assessed	10-12
	6b	Any changes to trial outcomes after the trial commenced, with reasons	
Sample size	7a	How sample size was determined	12
	7b	When applicable, explanation of any interim analyses and stopping guidelines	
Randomisation:			
Sequence generation	8a	Method used to generate the random allocation sequence	
	8b	Type of randomisation; details of any restriction (such as blocking and block size)	12
Allocation concealment mechanism	9	Mechanism used to implement the random allocation sequence (such as sequentially numbered containers), describing any steps taken to conceal the sequence until interventions were assigned	9

Implementation	10	Who generated the random allocation sequence, who enrolled participants, and who assigned participants to interventions	9
Blinding	11a	If done, who was blinded after assignment to interventions (for example, participants, care providers, those assessing outcomes) and how	
	11b	If relevant, description of the similarity of interventions	
Statistical methods	12a	Statistical methods used to compare groups for primary and secondary outcomes	12
	12b	Methods for additional analyses, such as subgroup analyses and adjusted analyses	
**Results**			
Participant flow (a diagram is strongly recommended)	13a	For each group, the numbers of participants who were randomly assigned, received intended treatment, and were analysed for the primary outcome	9 & Figure 1
	13b	For each group, losses and exclusions after randomisation, together with reasons	12
Recruitment	14a	Dates defining the periods of recruitment and follow-up	9
	14b	Why the trial ended or was stopped	
Baseline data	15	A table showing baseline demographic and clinical characteristics for each group	25
Numbers analysed	16	For each group, number of participants (denominator) included in each analysis and whether the analysis was by original assigned groups	24-28
Outcomes and estimation	17a	For each primary and secondary outcome, results for each group, and the estimated effect size and its precision (such as 95% confidence interval)	Figure 2A-2B
	17b	For binary outcomes, presentation of both absolute and relative effect sizes is recommended	14
Ancillary analyses	18	Results of any other analyses performed, including subgroup analyses and adjusted analyses, distinguishing pre-specified from exploratory	13
Harms	19	All important harms or unintended effects in each group (for specific guidance see CONSORT for harms)	
**Discussion**			
Limitations	20	Trial limitations, addressing sources of potential bias, imprecision, and, if relevant, multiplicity of analyses	17
Generalisability	21	Generalisability (external validity, applicability) of the trial findings	17

Interpretation	22	Interpretation consistent with results, balancing benefits and harms, and considering other relevant evidence	15-17
**Other information**			
Registration	23	Registration number and name of trial registry	12
Protocol	24	Where the full trial protocol can be accessed, if available	
Funding	25	Sources of funding and other support (such as supply of drugs), role of funders	2

*We strongly recommend reading this statement in conjunction with the CONSORT 2010 Explanation and Elaboration for important clarifications on all the items. If relevant, we also recommend reading CONSORT extensions for cluster randomised trials, non-inferiority and equivalence trials, non-pharmacological treatments, herbal interventions, and pragmatic trials. Additional extensions are forthcoming: for those and for up to date references relevant to this checklist, see www.consort-statement.org.
